# Raman Molecular Fingerprints of SARS‐CoV‐2 British Variant and the Concept of *Raman Barcode*

**DOI:** 10.1002/advs.202103287

**Published:** 2021-12-07

**Authors:** Giuseppe Pezzotti, Francesco Boschetto, Eriko Ohgitani, Yuki Fujita, Masaharu Shin‐Ya, Tetsuya Adachi, Toshiro Yamamoto, Narisato Kanamura, Elia Marin, Wenliang Zhu, Ichiro Nishimura, Osam Mazda

**Affiliations:** ^1^ Ceramic Physics Laboratory Kyoto Institute of Technology Sakyo‐ku, Matsugasaki Kyoto 606‐8585 Japan; ^2^ Department of Immunology Graduate School of Medical Science Kyoto Prefectural University of Medicine Kamigyo‐ku, 465 Kajii‐cho Kyoto 602‐8566 Japan; ^3^ Department of Orthopedic Surgery Tokyo Medical University 6‐7‐1 Nishi‐Shinjuku, Shinjuku‐ku Tokyo 160‐0023 Japan; ^4^ The Center for Advanced Medical Engineering and Informatics Osaka University 2‐2 Yamadaoka, Suita Osaka 565‐0854 Japan; ^5^ Institute of Biomaterials and Bioengineering Tokyo Medical and Dental University 2‐3‐10 Kanda‐Surugadai, Chiyoda‐ku Tokyo 101‐0062 Japan; ^6^ Department of Dental Medicine Graduate School of Medical Science Kyoto Prefectural University of Medicine Kamigyo‐ku Kyoto 602‐8566 Japan; ^7^ Division of Advanced Prosthodontics The Jane and Jerry Weintraub Center for Reconstructive Biotechnology UCLA School of Dentistry Los Angeles CA 90095 USA

**Keywords:** Raman, barcode, SARS‐CoV‐2, variants, fingerprints

## Abstract

The multiple mutations of the severe acute respiratory syndrome coronavirus 2 (SARS‐CoV‐2) virus have created variants with structural differences in both their spike and nucleocapsid proteins. While the functional relevance of these mutations is under continuous scrutiny, current findings have documented their detrimental impact in terms of affinity with host receptors, antibody resistance, and diagnostic sensitivity. Raman spectra collected on two British variant sub‐types found in Japan (QK002 and QHN001) are compared with that of the original Japanese isolate (JPN/TY/WK‐521), and found bold vibrational differences. These included: i) fractions of sulfur‐containing amino acid rotamers, ii) hydrophobic interactions of tyrosine phenol ring, iii) apparent fractions of RNA purines and pyrimidines, and iv) protein secondary structures. Building upon molecular scale results and their statistical validations, the authors propose to represent virus variants with a barcode specially tailored on Raman spectrum. Raman spectroscopy enables fast identification of virus variants, while the Raman barcode facilitates electronic recordkeeping and translates molecular characteristics into information rapidly accessible by users.

## Graphical abstract

Raman measurements clearly discriminate between SARS‐CoV‐2 variants and original isolate by resolving in times of tens of seconds conclusive differences in conformational isomerism of spike proteins. Key molecular information encrypted in the Raman spectrum is represented with a barcode locating the variant and decryptable into readable information through appropriate apps. The Raman barcode can support on‐site Coronavirus Disease 2019 (COVID‐19) virology diagnostics.

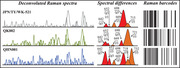

## 1 Introduction

SARS‐CoV‐2 continues to mutate in order to evade responses of the human immune system, and the mutations should be carefully monitored as they can reduce the effectiveness of vaccines and conceal themselves in diagnostic tests. Among the variants isolated so far, the British B.1.1.7 lineage shows a substantial transmission advantage over other lineages (i.e., 50–100% higher reproduction number).^[^ ^1^, ^2^, ^3^ ^]^ This transmission advantage, and the rapid increase in other strains with similar characteristics,^[^ ^4^, ^5^ ^]^ escalates the challenges in controlling the COVID‐19 pandemic. An official survey conducted recently by the Japanese National Institute of Infectious Diseases (JNIID) in Osaka and Hyogo Prefectures found that >80% of the cases reported by the beginning of May 2021 were from SARS‐CoV‐2 variants.^[^ ^6^ ^]^ The city of Kobe, in Hyogo Prefecture, recorded ≈70% of the reported cases of the British variant. The survey clearly indicates an increasing predominance of cases from SARS‐CoV‐2 British variants in the Kansai region. Another study recently published by the JNIID examined 803 infections caused by the SARS‐CoV‐2 British (B.1.1.7 lineage) variant found in Japan and estimated this variant as 1.32 times more infectious than the original Japanese isolate.^[^ ^7^ ^]^ However, the British variant may have been excluded from early data and so the estimated infectivity data could be lower than the real ones.

The point mutations so far developed in SARS‐CoV‐2 have been analyzed for protein coding genes,^[^ ^8^ ^]^ and genomic sequences of SARS‐CoV‐2 reference genomes deposited to a sequence database, which is continuously updated for nucleotides isolates collected from different hosts.^[^ ^9^, ^10^, ^11^ ^]^ High‐specificity detection of SARS‐CoV‐2 virions is usually achieved through real‐time reverse transcription‐quantitative polymerase chain reactions or serological enzyme‐linked immunosorbent assays. However, both methods are time‐consuming, fail to distinguish between the presence of virus and its inactivated state, and are incapable of detecting variants. The standard method adopted so far for detecting SARS‐CoV‐2 variants is next‐generation sequencing,^[^ ^12^ ^]^ which is also time‐consuming, expensive, and thus of limited utility in detecting and monitoring large‐scale variants. The above circumstances call for new testing platforms capable of detecting SARS‐CoV‐2 variants in a rapid and cost‐effective way.

In this study, we applied Raman spectroscopy to identify the SARS‐CoV‐2 British variants found in Japan as distinct from the original Japanese isolate. Upon preliminary statistical validations based on Pearson's correlation coefficient and using refined instrumentation and spectroscopic procedures, Raman spectra could be recorded in a time frame in the order of the tens of seconds. Clear differences were recorded between the spectra of variant sub‐types and that of the original isolate, thus proving the high sensitivity of the Raman approach in locating virus variants and related sub‐types. The Raman spectrum directly links to the molecular structure of the virions and provides a chemical fingerprint for different variants. This could be the missing key in on‐site virology diagnostics. A spectroscopic analysis based on a machine‐learning algorithm specifically crafted for the Raman spectrum is proposed here together with a *Raman barcode* approach to classifying different isolates. Our findings advance the state of the art of Raman spectroscopy in virology by presenting for the first time high‐resolution Raman spectra of the SARS‐CoV‐2 original isolate and the British variants, while proving the possibility of prompt on‐site discrimination among different viral variants through vibrational assessments.

## 2 Results

**Figure 1:**
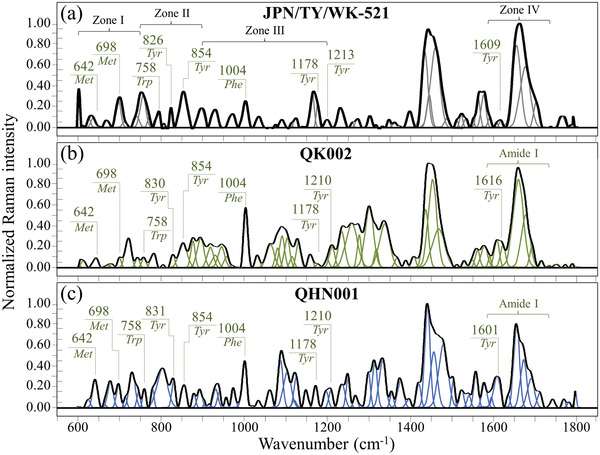
Raman spectra in the frequency interval 600–1800 cm^−1^ of a) the original Japanese isolate JPN/TY/WK‐521, b) variant QK002, and c) variant QHN001. Spectra are normalized with respect to the Amide I signal and deconvoluted into Gaussian‐Lorentzian band components according to Equation (m‐1) in Methods. Four Zones are emphasized in a) and labels show frequencies at maximum of selected bands (Met, Tyr, and Phe are abbreviations for methionine, tyrosine, and phenylalanine, respectively).

**Figure** **1** shows Raman spectra collected on the original Japanese isolate JPN/TY/WK‐521 (a), and two British variant sub‐types, QK002 (b), and QHN001 (c). An explanatory draft of the virus sample and sample/probe interaction is given in Figure M‐1 of Methods (Supporting Information). Four frequency intervals are shown and labeled as Zones I–IV at 600–750, 750–900, 900–1200, and 1600–1750 cm^−1^, respectively. The spectra were normalized with respect to their Amide II signal at 1460 cm^−1^, and deconvoluted into band components according to the algorithm given by Equation (m‐1) in Methods (Supporting Information). Frequencies at maximum and proposed vibrational origins are given in the Supporting Information (see Figure S1 and Tables S1a–d, Supporting Information). The spectra appear very different to each other, proving that Raman spectroscopy captured fundamental differences in the molecular structure of variants.

### 2.1 Rotamers of S‐Containing Amino Acids: Spectral Zone I

**Figure 2:**
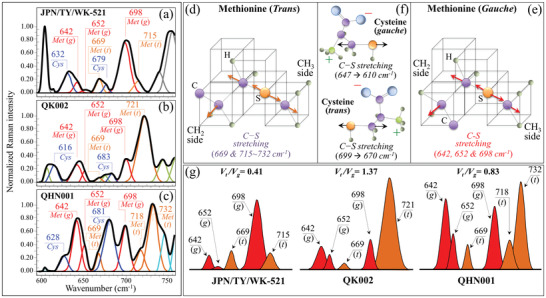
Low‐frequency Zone I (600–750 cm^−1^) of the Raman spectra of a) JPN/TY/WK‐521, b) QK002, and c) QHN001 viral strains; spectra are deconvoluted into a sequence of Gaussian‐Lorentzian sub‐bands (frequencies for selected bands shown in inset). The abbreviations Met and Cys refer to methionine and cysteine, respectively, while (t) and (g) locate trans and gauche rotamers, respectively. Structures and C—S stretching vibrational modes/frequencies of d) trans and e) gauche methionine rotamers; and, f), gauche and trans rotamers of cysteine and C—S stretching vibrational modes/frequencies. In (g), components are shown that represent signals from different rotameric configurations of methionine structure with related trans‐to‐gauche ratios, *V*_t_ /*V*_g_, ratios (given in inset together with labels of band frequencies and types of rotamer).

**Figure** **2**a–c shows the low‐frequency Zone I for the Raman spectra of JPN/TY/WK‐521, QK002, and QHN001 viral strains. This zone is dominated by vibrational signals relating to the C—S bond,^[^ ^13^ ^]^ which is only found in methionine and cysteine incorporated into viral proteins. The structures and main vibrational modes of the methionine and cysteine rotamers are shown in Figure 2d–g (see labels). Reported substitutions in the spike‐protein amino acid sequence of the British variants do not mention methionine or cysteine but only spell asparagine‐to‐tyrosine and aspartate‐to‐glycine substitutions.^[^ ^14^, ^15^ ^]^ However, high‐resolution spectra in Figure 2 show significant differences in the fractions of S‐containing amino acid rotamers between the two British variant sub‐types and the original Japanese isolate, and between the variant sub‐types themselves. The Raman spectrum is very sensitive to molecular symmetry, which appears as pronounced spectral differences. This characteristic, coupled with the high sensitivity of the Raman spectrum to C—S bonds, reveals the distinctive structural details of the S‐containing molecules in SARS‐CoV‐2 viral strains. Figure 2d,e show *trans* and *gauche* methionine rotamers and their related C—S stretching modes,^[^ ^16^, ^17^ ^]^ C—S stretching bands from methionine residues are found at 642, 652, 669, 698, and 715–732 cm^−1^ (see Figure 2). Bands at 642 and 652 cm^−1^ represent C—S bond stretching vibrations on the CH_2_ side of molecules in *gauche* rotameric configuration, while the signal at 669 cm^−1^ arises from the same vibrational mode on the CH_2_ side for molecules in *trans* configuration. The signal at ≈642 cm^−1^ incorporates contributions from tyrosine (ring deformation mode).^[^ ^18^ ^]^ Conversely, signals at 698, and 715–732 cm^−1^ relate to C—S stretching on the CH_3_ carboxyl side in *gauche* and *trans* configurations (see Table S1a, Supporting Information).^[^ ^16^, ^17^ ^]^

Methionine is one of the most hydrophobic amino acids present in nature, therefore, its residues are generally located at buried sites in the hydrophobic core of globular proteins, or in links with lipid bilayers in membrane‐spanning protein domains. Accordingly, the frequencies of the C—S bands are not expected to show any marked dependence on environmental pH. However, the molecular symmetry characteristics of methionine residues of the spike protein play a key‐role in a number of functions during the virus lifetime^[^ ^19^, ^20^ ^]^ Importantly, different SARS‐CoV‐2 isolates possess different fractions of methionine rotamers, a consequence of the different amino acid sequences to which methionine links. Different *trans*‐to‐*gauche* ratios, *V*_t_ /*V*_g_, could be computed for different viral strains (0.41, 1.37, and 0.83, for JPN/TY/WK‐521, QK002, and QHN001 isolates; see labels in Figure 2g). These different values reflect the different chirality of S‐containing amino acids, peculiar to individual strains and are efficient in variant recognition.

The zwitterionic structure of monoclinic cysteine rotamers and the expected frequencies for their C‐S stretching mode in molecules with different chirality characteristics are shown in Figure 2f.^[^ ^21^, ^22^, ^23^, ^24^ ^]^ Unlike methionine, the thiol side chain in cysteine participates as a nucleophile in environmental reactions and is susceptible to oxidation. C‐S stretching bands are expected at ≈630, and ≈670 cm^–1^.^[^ ^21^, ^22^, ^23^, ^24^ ^]^ We indeed observed C‐S bands at ≈670 cm^–1^ (distinct from those of methionine) in all investigated strains. Signals at 632 and 628 cm^–1^ were observed in the JPN/TY/WK‐521 isolate and QHN001 variants, respectively (see labels in Figure 2), while only a band of cysteine located at 616 cm^–1^ (COO^−^ rocking)^[^ ^24^ ^]^ (missing in JPN/TY/WK‐521 and QHN001) could be observed in the QK002 variant. This signal senses different pH environments at the virion surface, as discussed later.

### 2.2 Tyrosine Phenol Ring as a Sensor of Interface pH: Spectral Zone II

**Figure 3:**
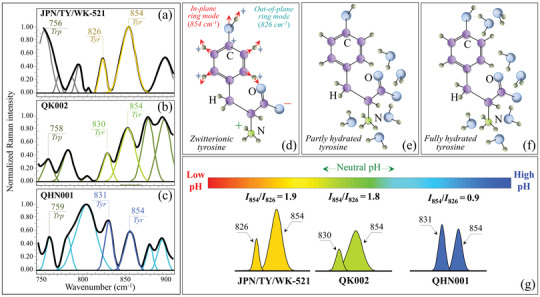
Spectral Zone II (750–900 cm^−1^) of the Raman spectra of a) JPN/TY/WK‐521, b) QK002, and c) QHN001 viral strains; spectra are deconvoluted into a sequence of Gaussian‐Lorentzian sub‐bands (frequencies for selected bands shown in inset). The abbreviations Trp and Tyr refer to tryptophan and tyrosine, respectively. Structure of tyrosine in d) zwitterionic, e) non‐hydrated, and f) fully hydrated condition are shown together with in‐plane and out‐of‐plane vibrational modes of the phenol ring according to Ref. ^[27]^. In (g), components are shown from which the Raman ratio, *I*_854_/*I*_826_, together with the expected values of environmental pH at the surface of different SARS‐CoV‐2 viral strains (see labels in inset).

The important feature found in Zone II (**Figure** **3**) relates to the intensity ratio of two bands of tyrosine (Fermi doublet) located at 854 and 826 cm^–1^ (spectra from JPN/TY/WK‐521, QK002, and QHN001 isolates in (a), (b), and (c)). This ratio, referred to as *I*_854_/*I*_826_, is diagnostic of the H‐bonding environment around tyrosine units, the lower the ratio the more hydrophobic the environment in which the tyrosine residue is embedded.^[^ ^25^, ^26^, ^27^ ^]^ According to Hernandez et al.,^[^ ^27^ ^]^ the components of the tyrosine doublet originate from two independent vibrational modes of the phenol ring, namely, in‐plane ring breathing (854 cm^–1^) and out‐of‐plane C‐H bending (826 cm^–1^) (see Table S1b, Supporting Information). The *I*_854_/*I*_826_ ratio is a sensor of the hydrophobic/hydrophilic balance in environmental interactions at the virion surface. A low *I*_854_/*I*_826_ ratio represents a hydrophobic tyrosine configuration, tyrosine being most hydrophobic in an alkaline environment, and vice versa for an acidic environment. The different structures of tyrosine are shown in Figure 3d (zwitterionic), (e) (non‐hydrated), and (f) (fully hydrated) together with in‐plane and out‐of‐plane vibrational modes of the phenol ring.^[^ ^27^ ^]^ Figure 3g shows the relationship between the Raman ratio, *I*_854_/*I*_826_, and environmental pH, as detected for different SARS‐CoV‐2 viral strains. The *I*_854_/*I*_826_ intensity ratio experienced a maximum value in the spectrum of the JPN/TY/WK‐521 isolate (1.9), while the ratios found for the QK002, and QHN001 variant sub‐types were 1.8, and 0.9, respectively. The above values suggest that the British variants have shifted from the acidic environment of the original isolate toward neutral‐to‐alkaline surface protonation conditions. Viruses possess a pH‐dependent surface charge in polar media (e.g., water). This electrostatic charge is key in their mobility and governs their sorption processes. According to a previous study,^[^ ^28^ ^]^ we assigned the character of a strong hydrogen‐bond donor to the tyrosine phenoxyl proton in the QHN001 isolate, while the tyrosine phenoxyl oxygen in both JPN/TY/WK‐521 and QK002 virions behave as a strong hydrogen‐bond acceptor. In other words, the tyrosine doublet ratio can be taken as a marker of the virion/environment equilibrium in aqueous chemistry. Again, this characteristic clearly differed for different SARS‐CoV‐2 viral strains.

### 2.3 “Raman Fractions” of RNA Bases: Spectral Zone III

**Figure 4:**
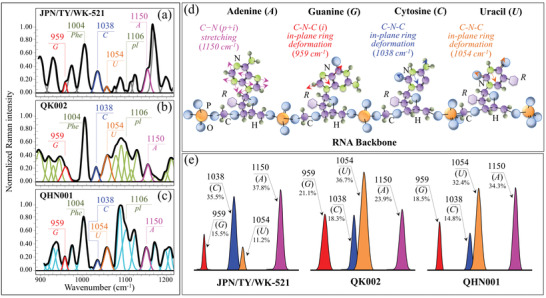
Spectral Zone III (900–1200 cm^−1^) of the Raman spectra of a) JPN/TY/WK‐521, b) QK002, and c) QHN001 viral strains; spectra are deconvoluted into a sequence of Gaussian‐Lorentzian sub‐bands (frequencies for selected bands shown in inset). The abbreviations G, C, U, pl, and A refer to guanine, cytosine, uracil, phosphodiester linkages, and adenosine, respectively. d) Schematic draft of purines and pyrimidines with phosphodiester linkages together with the ring vibrational modes selected to locate different RNA bases; in (e), signals are shown, which are used to estimate the fractions of different purine and pyrimidines (shown in inset together with the frequencies of the selected signals) found in different strains. The abbreviations *p* and *i* (in brackets in (d)) refer to pyridine and imidazole rings, respectively.

**Figure** **4**a–c show Zone III Raman spectra for the JPN/TY/WK‐521 original isolate, and the QK002 and QHN001 sub‐types (labeling and vibrational origins in Figure S1 and Table S1c, Supporting Information). Besides containing the main Raman signal of phenylalanine at 1004 cm^−1^ (symmetric ring breathing), this spectral zone incorporates ring‐related signals from individual RNA purines and pyrimidines. Both cytosine (*C*) and uracil (*U*) pyrimidines presented isolated bands from C‐N‐C in‐plane deformation of their heterocyclic aromatic ring centered at 1038 and 1054 cm^−1^.^[^ ^29^, ^30^, ^31^ ^]^ The same type of vibration appeared in the guanine (*G*) purine at 959 cm^−1^, while a cumulative signal from C–N stretching modes in both imidazole and pyridine rings of the adenine (*A*) purine was found at 1150 cm^−1^.^[^ ^31^, ^32^, ^33^ ^]^ Note that the above signals could clearly be detected in the spectra of all strains, although purine signals appeared as shoulder bands in the spectrum of the QK002 variant. Figure 4d shows schematic drafts of purines and pyrimidines in the RNA sequence together with their selected vibrational fingerprints.

Fingerprint signals were selected to minimize overlap with signals from different molecules, according to the machine‐learning algorithm described in the Supporting Information. The relative intensity ratio of selected fingerprints bands from purines and pyrimidines in Zone III showed quite different values for different strains (see spectra in Figure 4). In Figure 4e, the intensity differences of fingerprint signals are schematically drawn for different strains, together with their fractional intensity values (computed by assuming as 100% the sum of selected signals from all purine and pyrimidine bases). Remarkably, the sum of the fingerprint Raman intensities of different bases was the same for the two different variant sub‐types (i.e., within an experimental scatter of ±1%), but it was much larger than that found in the original Japanese isolate. This suggests that “reshuffling” of genome components occurred differently in the two variants, and that both variants underwent significant alteration of the total amount of RNA genome as compared to the original isolate. In viruses, the band at 1106 cm^−1^ represents phosphodiester linkages (*pl*) in the RNA backbone (see spectra in Figure 4 and Table S1c, Supporting Information).^[^ ^34^ ^]^ The intensity of this band is proportional to the number of nucleotides units of ordered structures and, consequently, to the number of nucleotides involved in secondary interactions, and accordingly, both variants presented a much higher probability of secondary interaction as compared to the original isolate. Labels in the inset of Figure 4e give the computed fractions of individual bases. It should be emphasized that the fractions computed from the intensities of the Raman bands do not represent the actual fractions computed by genome analyses (see comparison between fractions computed by Raman and genome analyses in Table S2, Supporting Information). Unlike conventional genome analyses, base pairing influences the computed “Raman fractions”. However, this characteristic appears to enhance the differences between different isolates, and it is useful to distinguish variants. The segmented composition of influenza viral genomes promotes reassortment; however, not all permutations of segment reassortment occur at the same frequency, with certain reassortment events being observed at higher rates.^[^ ^35^ ^]^ Since viral nucleoproteins associate at preferential RNA sites,^[^ ^36^ ^]^ variations in genome composition are linked to changes in the secondary structure of proteins, as discussed below.

### 2.4 Protein Secondary Structure: Spectral Zone IV

**Figure 5:**
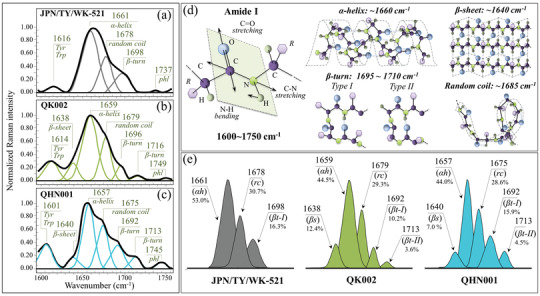
Spectral Zone IV (Amide I, 1600–1750 cm^−1^) of the Raman spectra of a) JPN/TY/WK‐521, b) QK002, and c) QHN001 viral strains; spectra are deconvoluted into a sequence of Gaussian‐Lorentzian sub‐bands (frequencies for selected bands shown in inset). The abbreviations Trp and Tyr refer to tryptophan and tyrosine, respectively. d) Schematic drafts of the Amide I vibrational mode, the different secondary structure of proteins and related frequencies; in e), signals are shown, which are used to estimate the fractions of different protein secondary structures (shown in inset together with the frequencies of the selected signals) found in different strains. The abbreviations *β*s, *α*h, rc, and *β*t‐I, *β*t‐II, and phl represent *β*‐sheet, *α*‐helix, random coil, two types of *β*‐turn rotamers, and phospholipids, respectively.

In **Figure** **5**a–c, deconvoluted Raman spectra in the Amide I frequency region (1600–1750 cm^−1^) are shown for the original JPN/TY/WK‐521 Japanese isolate, and the two British variant sub‐types QK002, and QHN001 (see labels). Deconvoluted Amide I band components, centered at 1638–1640, 1657–1661, 1675–1679, 1692–1698, and 1713–1716 cm^−1^, were assigned to *β*‐sheet (*βs*), *α*‐helix (*αh*), random coil (*rc*), and *β*‐turn rotamers (*βt‐I* and *βt‐II*) (see Figure S1 and Table S1d, Supporting Information).^[^ ^36^ ^]^ Figure 5d shows schematic drafts of the secondary structures of proteins and their expected ranges of vibrational frequencies. In Figure 5e, Amide I signals are compared for spectra recorded different isolates (see labels). Despite a common feature in the predominance of *α*‐helix component in all strains, the Amide I spectra of the British variants showed clearly different morphologies. The differences reflected substantial dissimilarity in the protein secondary structure of the different strains. The JPN/TY/WK‐521 original isolate lacked signals from *β*‐sheet and *β*‐turn Type II rotamer, which instead appeared in both British variant sub‐types. The fractional amounts of different protein structures computed from relative intensities of the Amide I spectrum are given in the inset of Figure 5e. According to Eker et al.,^[^ ^37^ ^]^ a hydrophobic environment leads to a propensity to form *β*‐sheet. Our results corroborate this, since the two British variant sub‐types, which newly included the *β*‐sheet structure, also experienced a lowered *I*_854_/*I*_826_ tyrosine ratio (i.e., a marker of increased environmental hydrophobicity) as compared with the original Japanese isolate. The conformational preference of viral proteins has key implications in peptide immunogenicity and can be swiftly captured by Raman spectroscopic assessments in the Amide I zone.

## 3 Discussion

### 3.1 Importance of the Raman Technology in the COVID‐19 Pandemic

Regional surveys by the Japanese Government showed a surge in the British coronavirus variants with N501Y mutation in the Kansai area in the first half of 2021, and indicate their possible increased transmissibility as compared to the original isolate. Insurgence of new variants is already occurring and will likely continue in the near future in countries with a low percent of vaccinated population. Our Raman analysis discovered bold differences in signals from RNA bases in both QK002 and QHN001 British variant sub‐types. The Raman fractional data did not fully correspond to the genomic data of the variants. However, they represented a new set of “Raman genomic” data distinctive of the variants. Once implemented into a vibrational library, the “Raman genome” could provide insightful and promptly obtainable information as compared to conventional genomic analyses. RNA sequencing has a wide‐ranging impact from diagnosis and pathogenesis to vaccine design and viral ecology.^[^ ^38^ ^]^ This study shows for the first time the possibility to use, on‐site and in real time, the unique sensitivity of Raman spectroscopy in locating molecular rotamers, protonation conditions of the viral surface, genomic differences, and protein secondary structures for discriminating between different variants of the SARS‐CoV‐2 virus. This study is in line with our previous studies of variants of Influenza virus.^[^ ^39^ ^]^ The Raman characteristics yield molecular‐scale information uniquely discriminating among different variants. Facilitating real‐time data access and exchange is an essential step in tracking variants and in estimating their spread and evolutionary mutation rate. Raman technology could provide an invaluable contribution to the presently available practices of traceability, tracking, and record‐keeping of viral spread.

Important aspects of the present work are the spectroscopic identification of different variants and the observation of spectroscopic differences between SARS‐CoV‐2 viral sub‐types. With the COVID‐19 pandemic rapidly expanding, the fast evolution of variants into viral sub‐types may be difficult to monitor and standardize. From a purely virological point of view, the discovery of multiplication of sub‐types within the same variant is an unequivocal proof that the so‐called “founder effect” plays a role at least as important as that of selection pressure. In other words, the competitive advantage of a given variant with respect to viral replication, transmission, or escape from immunity overlaps locally inherited genomic mutations. As a result, a variant sub‐type might survive regardless of its actual viral fitness. As widely recognized, it is the interplay of natural selection and chance events that defines virus evolution in local communities and leads to the generation of a number of variants and their sub‐types. In this context, we emphasize that it is both a remarkable and fortunate circumstance that Raman spectroscopy could distinguish among virus sub‐types by virtue of its high sensitivity to molecular symmetry, base pairing, and protein secondary structures. This property of the Raman method could, in principle, allow geographical maps of viral outbreaks in downstream analyses (to support or even replace real‐time tracing), and plotting spatiotemporal visualization of viral dynamics. We anticipate here the possibility to detect additional sub‐types for the British variant as well as for South African, Brazilian, and Indian variants, according to our preliminary unpublished studies.

From a purely structural point of view, the fractional balance between the two S‐containing amino acids, methionine, and cysteine, in the protein structure of virion sub‐types should conceivably be the result of genomic interplay with the cellular environment in which the specific viral sub‐type was developed. Note also that the catalytic and enzymatic activity of cysteine residues bound by disulfide bridges is key in both protein folding and stability; thus, its abundance directly links to viral fitness. The above two arguments reasonably justify the existence of a variety of virus sub‐types with different methionine/cysteine fractions as modulated by both genomic and founder‐effect‐driven kinetics.

### 3.2 Benefits of Electronic Bar Coding as a Measure of Pandemic Control

**Figure 6:**
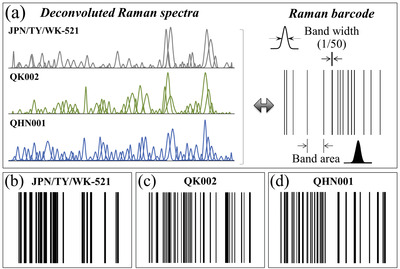
a) Sequences of Raman Gaussian‐Lorentzian bands are shown as deconvoluted from average Raman spectra recorded on the original Japanese isolate and two British variants (see labels); and, algorithm to convert a band sequence into a barcode. In (b–d), barcodes are shown that are constructed for the sub‐band sequences in (a).

In view of the present findings, we propose to use a barcode with the Raman spectrum of different variants and their sub‐types in order to enable efficient electronic recordkeeping and increase users’ accessibility to the emergence and transmission characteristics of variants through apps and user‐friendly software. Barcodes could be matched to the Raman spectrum in a number of different ways, and provide the Raman spectroscopic method with the flexibility and the swiftness necessary to inform users about viral mutations periodically developed under different local conditions. Examples of Raman spectrum/barcode matching are given in **Figure** **6**. In Figure 6a, sequences of Raman Gaussian‐Lorentzian bands are shown as deconvoluted from average Raman spectra recorded on the original Japanese isolate and two British variant sub‐types. An algorithm is also shown, which converts the band sequence into a barcode by assigning to each band a line with thickness equal to 1/50 of the sub‐band width and a distance from the successive line proportional to the band area. The “Raman barcodes” unambiguously locate the virus variants/sub‐types (Figure 6b) and can be decrypted into easily readable information through appropriate apps.

The use of electronic bar coding has already been set in practice in a number of hospitals and health systems in order to reduce medication errors. The increasing popularity of this technology arises from the need to add an additional level of verification into medication administration processes.^[^ ^40^ ^]^

Also in the context of COVID‐19 pandemic, a “genetic barcode” has been proposed, which uses information from a global database of genetic data extracted from coronavirus tests.^[^ ^41^ ^]^ Similar to the “genetic barcode”, the “Raman barcode” is capable to locate the presence of a multiplicity of slightly different versions of the virus among infected people as sub‐types within their general classification into individual variants. Such details could become of fundamental importance in future management of the pandemic because small genetic differences could be key in future drugs and vaccine developments. Moreover, the Raman barcode technology could be useful in tracking whether new cases of infection are the result of local evolution/transmission or come from different parts of the world. The “Raman barcode” shares with the “genetic barcode” the capacity of thoroughly locating genetically significant virus sub‐types of individual variants, but might surpass it in swiftness and flexibility regarding on‐site diagnostics. In summary, the authors believe that the introduction of the “Raman barcode” approach into capillary networks would be an important contribution to the real‐time management of information concerning virus evolution during pandemics. In the immediate future, once automatically linked to clouds, it could report on‐site how the virus is evolving under the selective pressure of the polyclonal immune response in vaccinated people.

### 3.3 Statistical Assessments and Limitations of the Present Study

In order to validate the clear differences in average spectra detected among different variants/sub‐types (see Figure 1), which we used here in discussing differences at the molecular scale, we also attempted a statistical evaluation of the spectral data collected on different sub‐types of the British variant as compared to the original Japanese isolate (see statistical assessments in the Supporting Information). An approach based on the Pearson's correlation coefficient^[^ ^42^ ^]^ (PC), *r*, was adopted, according to the following considerations:

i) The PC value has widely been used for pixel‐to‐pixel image correlations and to compare similarities between Raman spectra considered as strings of intensity values detected for different CCD pixels.^[^ ^43^ ^]^
ii) Among the available statistical methods, the one based on PC appears to be most suitable to assess real on‐site situations, in which a single spectrum collected on‐site from a patient, needs to be matched with a multitude of average spectra from a library.^[^ ^44^ ^]^

The PC algorithm was designed to select the best matching spectrum based on a similarity criterion (see mathematical formulation in Equation (S1), Supporting Information), with *r* = 1 stemming for perfectly identical spectra and *r* = 0 indicating a complete mismatch. The results of the PC‐based statistical analysis performed in this study are summarized in Table S3, Supporting Information, in terms of the *r* values and related standard deviations. A cross comparison of *r* values among variants and sub‐types systematically confirmed an almost identical pattern (*r* > 0.920) when comparing spectra collected on samples of the same variant/sub‐type, while low PC scores (*r* < 0.420) were retrieved for any mixed matching among different variants. All statistical matches of spectra collected on the same variant presented a quite small standard deviation (i.e., in the order of 10^−2^), while matching spectra from different variants involved larger standard deviations (in the order of 10^–1^) in addition to low *r* values (see Table S3, Supporting Information). An interesting result was that cross‐matches between different sub‐types of the British variant gave high *r* values (i.e., comparable with those recorded for spectra collected on the same sub‐type), but low standard deviations (i.e., comparable with those recorded for different variants). In order to support the statistical analysis discussed above, comparisons between average Raman spectra and spectra collected at individual locations on the same variant/sub‐type were made, as shown in the Supporting Information (from Figures S2–S11, Supporting Information). Related spectroscopic analyses are given for fractions of sulfur‐containing amino acid rotamers, tyrosine phenol ring, apparent fractions of RNA purines and pyrimidines, and protein secondary structures. Individual spectra collected at different locations generally showed agreement with the corresponding average one in agreement with Pearson's statistical assessments. In particular, sub‐types of the British variant were closer to their respective average spectra (PC = 0.985 ± 0.027 and 0.996 ± 0.001 for QHN001 and QK002, respectively) as compared to the matching shown by the original Japanese isolate JPN/TY/WK‐521 with its average spectrum (PC = 0.925 ± 0.065) (see Table S3, Supporting Information). However, some anomalous spectra were locally found with interesting spectral variations. Such anomalous spectra, although having no statistical significance, unveiled some interesting structural features. These additional aspects are discussed in the Supporting Information.

Although the present statistical assessment should only be considered as a preliminary one, it strengthens our thesis of high sensitivity for a Raman approach in SARS‐CoV‐2 viral speciation. While mathematical methods remain to be rigorously tested and standardized to confirm the elimination of any eventual influence of spectral background and signal‐to‐noise ratio, we consider the present results as quite encouraging. We have suggested refined procedures of spectral pre‐treatments in the Supporting Information, which should critically be evaluated in future works. There are additional points to be assessed in future evaluations. First, spectroscopic experiments on samples from on‐site patients should be performed to confirm the possibility of matching them with high reliability to average spectra from a library. Note, in this context, that the different variants/sub‐types investigated in this study were all obtained from the Japanese National Institute of Infectious Diseases after a rigorous classification through genomic analyses based on a large number of biological replicates. Second, a precise spectroscopic scrutiny should be made of the in vitro process of viral propagation, which we applied in the present study for achieving a fixed titer concentration from the small fractions of virions received per each variant/sub‐type, in order to check whether such process could have any influence on the original virus identity through additional interplay between genome‐driven mutations and chance events.

Despite the above limitations, the present spectroscopic study shows for the first time the existence of significant molecular gradients in the population of SARS‐CoV‐2 virion structures belonging to different variants/sub‐types. Similar to humans, such “somatic” differences ultimately cluster into well‐defined average patterns with high statistical reliability.

## 4 Conclusion

In conclusion, we showed that Raman analyses could provide virologists with insightful and rapid information on SARS‐CoV‐2 variants and their sub‐types through a clear view on virus structure at the molecular scale. The high sensitivity of Raman spectroscopy in virus speciation and the importance of molecular symmetry in SARS‐CoV‐2 virus classification could also be exploited in the case of other viral strains and pathogenic species. Data obtained from Raman measurements are particularly informative regarding the molecular details of conformational isomerism in viral proteins, fractional amounts of RNA purine and pyrimidine bases, protonation conditions of the viral surface, and protein secondary structures. The Raman spectrum encrypts the solvent‐dependent structural propensity of viral proteins, directly linking to site‐specific interactions with the key molecular subgroups that define the viral morphogenetic pathway. While the construction of a wide Raman library of SARS‐CoV‐2 variants/sub‐types is presently ongoing, it is hoped that the experimental evidence presented here will provide impetus for developing high‐sensitivity portable Raman equipment for fast on‐site characterizations of virus variants.

## Conflict of Interest

The authors declare no conflict of interest.

## Supporting information

Supporting Information

## Data Availability

The data that support the findings of this study are available from the corresponding author upon reasonable request.
