# Supplementary material for: Raman Molecular Fingerprints of SARS‐CoV‐2 British Variant and the Concept of Raman Barcode
Source: Adv Sci (Weinh). 2021 Dec 7;9(3):2103287. doi: 10.1002/advs.202103287 (PMC8787433; doi:10.1002/advs.202103287)
Supplement: Supplementary file 1 — Supporting Information [file ADVS-9-2103287-s001.pdf]

ADVANCED SCIENCE
Open Access

Supporting Information

for *Adv. Sci.*, DOI: 10.1002/advs.202103287

Raman molecular fingerprints of SARS-CoV-2 British variant and the concept of *Raman barcode*

Giuseppe Pezzotti,[1,2,3,4,5] Francesco Boschetto,[1,2] Eriko Ohgitani,[2] Yuki Fujita,[1] Masaharu Shin-Ya,[2] Tetsuya Adachi,[6] Toshiro Yamamoto,[6] Narisato Kanamura,[6] Elia Marin,[1,6] Wenliang Zhu,[1] Ichiro Nishimura,[7] and Osam Mazda[2*]*

Methods

M-1. Preparation of SARS-CoV-2 variants for Raman analysis

SARS-CoV-2 virus experiments were conducted in a biosafety level 3 (BSL-3) bio-containment facility using BSL-3 work practices.

SARS-CoV-2 viral stocks of the original Japanese isolate (JPN/TY/WK-521) and of two British variants (betacoronavirus type B.1.1.7 lineage) found at airport quarantine station in Japan (hCoV-19/Japan/*QK002*/2020 and hCoV-19/Japan/QHN001/2020; henceforth referred to as QK002 and QHN001, respectively) were obtained from the Japanese National Institute of Infectious Diseases after detailed genomic characterizations on a series of biological replicates as also reported in Section S-2 of the Supplementary Information. Further details are, as follows:

Materials

Viruses: SARS-CoV-2 strain QK002 and strain QK002 supplied by National Institute of Infectious Diseases (Japan).

Cells: VeroE6/TMPRSS2 purchased from JCRB Cell Bank (VeroE6 cells expressing the transmembrane serine protease TMPRSS2).

Growth medium, GM: Dulbecco's modified Eagle's minimum essential medium (DMEM) (NACALAI TESQUE, INC., Kyoto, Japan) supplemented with G 418 disulfate aqueous solution (1 mg/ml), penicillin (100 units/ml), streptomycin (100 lg/ml) and 5% fetal bovine serum.

Maintenance solution, MS: Phenol red free Dulbecco's modified Eagle's minimum essential medium (DMEM) supplemented with penicillin, streptomycin and 0.5% fetal bovine serum.

Glass bottom microwell dish: MatTek Corporation, Ashland, MA, USA.

Procedures

VeroE6/TMPRSS2 cells were grown in GM and maintained at 37 °C in an atmosphere of 5 % CO_2.

Viruses were infected at Multiplicity of infection (MOI) of 0.01 and propagated in the VeroE6/TMPRSS2 cells at 37 °C for 2 days. Each 90 ml of the culture supernatants were harvested and centrifuged at 4800 rpm for 20 min. The clarified supernatants were assayed viral infectivity titers using TCID50 (Median Tissue Culture Infectious Dose method).

Titer

JPN/TY/WK-521: 3.4 x 10^7 $TCID_{50}$/ml

QK002: 3.4 x 10^7 $TCID_{50}$/ml

QHN001: 4.8 x 10^7 $TCID_{50}$/ml

The supernatants were concentrated 1/15 using ultrafiltration membrane, Amicon Ultra-15 Centrifugal Filter Unit (Merck, Darmstadt, Germany).

The concentrates were suspended in PBS (-) and ultracentrifuged at 24000 rpm, 3 h at 4 ºC. After removing the supernatants, the pellets were resuspended in 50 µl of PBS (-), put on glass bottom dishes, and air-dried. After fixation with 4% paraformaldehyde for 20 min at R.T. and washing with distilled water twice, the pellets were air-dried again. They were kept in dry environment at 4 °C until Raman analysis was performed.

The following concentrations of virus titers were on the glass bottom at the time of Raman measurement:

JPN/TY/WK-521: 3.4 x 10^7 x 90 = 3.1 x 10^9 $TCID_{50}$

QK002: 3.4 x 10^7 x 90 = 3.1 x 10^9 $TCID_{50}$

QHN001: 4.8 x 10^7 x 90 = 4.3 x 10^9 $TCID_{50}$

The area of the glass bottom (7 mm in diameter) was 38.5 mm^2.

M-2. Raman spectroscopy

A Raman spectrometer (LabRAM HR800, Horiba/Jobin-Yvon, Kyoto, Japan) was used for *in situ* spectral analysis of different viral strains. The spectroscope, which was set in confocal mode, used a holographic notch filter to concurrently provide high-efficiency and high-resolution spectral acquisitions. Two different wavelengths were used for the incoming light: 532 nm and 785 nm solid-state laser source operating in both cases at 10 mW. The spectrum of a sample only consisting of paraformaldehyde was preliminary recorded under both green and red lights using exactly the same spectroscopic measurement conditions. Under red light, the paraformaldehyde used for fixation only appeared as a broad background, which could easily be eliminated according to a standardized procedure, as explained later in this section. Spectra with the above two different wavelengths were collected and compared. A spectral resolution of ~1 cm^{-1} was achieved upon analyzing the Raman scattered light by a double monochromator connected with an air-cooled charge-coupled device (CCD) detector (Andor DV420-OE322; 1024 × 256 pixels); the grating used in the spectrometer had the high resolution of 1800 gr/mm. The acquisition time of a single spectrum was typically 10 s. However, several consecutive acquisitions were made at the same spot to minimize noise. The laser spot was ~2 µm as focused on the sample through a 50x optical lens. In collecting average spectra, tens of spectra were col-

lected at different locations over areas of ~2 mm^2 for each type of sample and averaged. A schematic draft explaining the sample setup and the sample/probe interaction is given in the figure below.

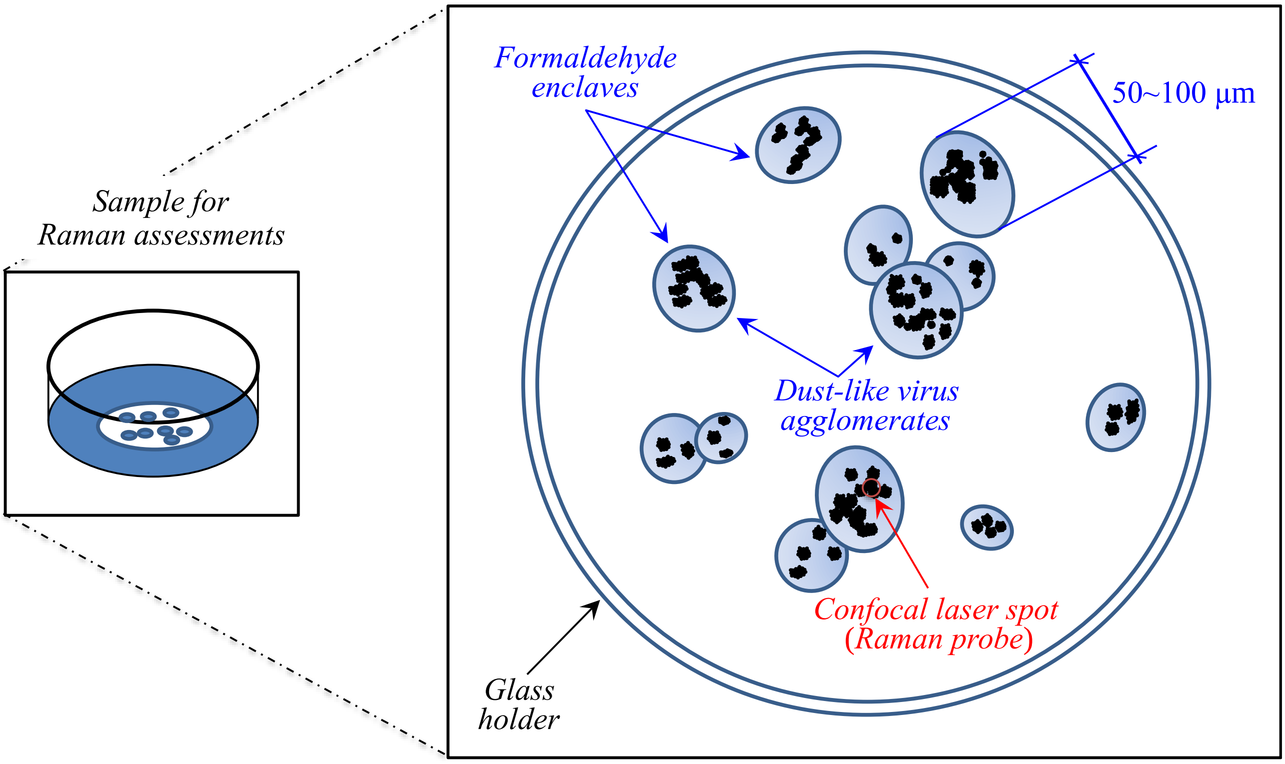

Fig. M-1: Schematic of the sample setup and sample/probe interaction.

As seen in the above draft, agglomerations of virions were spotted into semi-ellipsoidal pools (enclaves) of paraformaldehyde typically 50~100 μm in diameter. Under the microscope, the agglomerated virions appeared as black/grey "dust" with variable focal positions. Accordingly, the micrometric laser spot was focused at selected locations of strong signal to collect the Raman spectrum. A preliminary *z*-scan was needed to focus the laser exactly on the virions and to maximize their spectrum with respect to the formaldehyde broad background. Spectra were collected on different enclaves and averaged for each sample.

In pre-treating the collected Raman spectra, baseline subtraction was preliminary optimized and standardized according to a comparison between three different methods: polynomial fitting,[1] asymmetric least square method,[2] and penalized spline smoothing based on vector transformation.[3] In background removal by polynomial fitting,[1] the background was fitted to a low-order polynomial by iteratively determining the polynomial parameters that minimize a least-square criterion. This method is

time-consuming but is conceptually the simplest one, being quite widely used in Raman spectroscopy. Other options exist when baseline drifts in Raman spectra mainly arise from a fluorescence noise, generating a broad background that overlaps with the Raman spectrum. This was indeed the case here, due to the presence of the overlapping broad but otherwise featureless spectrum of paraformaldehyde. However, baseline shifts differed at different locations or even at the same location when different wavenumber intervals were selected. Baselines were either slightly or severely drifting, and the spectral preprocessing was optimized to be insensitive to the noise morphology. In the present Raman experiments, algorithms built to reproduce those proposed in Refs. 2 and 3 appeared both suitable and reliable for Raman analyses.

The collected Raman spectra were treated with a baseline subtraction procedure and automatically deconvoluted into series of Voigtian sub-bands using commercial software (LabSpec 4.02, Horiba/Jobin-Yvon, Kyoto, Japan). In deconvolutive spectral analyses, we applied a machine-learning approach using an in-house built automatic solver, $S_{av}(\nu)$, exploiting a linear polynomial expression of Voigtian functions, $V(\Delta\nu, \sigma, \gamma)$, where ν, $\Delta\nu$, σ, and γ represent the Raman frequency, the shift in frequency from each sub-band's maximum (ν_0), the standard deviation of each Voigtian component, and the full-width at half-maximum of the Lorentzian component, respectively. An algorithm searching for the minimum value of the difference between the experimental and the fitted spectrum was then set, as follows:

$$S_{av}(\nu) - \Sigma_i \alpha_i \Sigma_j \beta_{ij} V_{ij}(\nu_0, \Delta\nu, \sigma, \gamma) \cong 0 \qquad \text{(m-1)}$$

where the index i locates each compound in a series of n compounds contributing to the overall spectrum, and the index j locates each Voigtian sub-band of a series of m compounds in the Raman spectrum of each compound of an n series. A computer program was set to optimize the above algorithm after choosing a series of Voigtian sub-bands from the deconvoluted spectra of pre-selected compounds included in a database of key biomolecules in aqueous solution and in solid state according to the chemical and structural peculiarities of virions. A pre-selection was made according to the literature, from spectra collected in aqueous solution. After picking up spectral sub-bands of elementary compounds from the library, the algorithm pinpointed the closest matches to the experimental spectra according to the following criteria: (i) preserving relative intensities (β_{ij}), (ii) assigning spectral positions (ν_0), and full-width

at half-maximum (σ and γ) values for specific sub-bands from each elementary compound within ±3 cm^{-1} (i.e., to include the possibility of alterations of the molecular structure in the virion structure). The conditions imposed on band positions, relative intensity, and bandwidths provided the required mathematical constraints to univocally deconvolute the experimental spectra. The computational work produced two outcomes: (i) spectra could be screened automatically and an appropriate deconvolution be suggested by finding the closest match with the experimental spectrum through Eq. (m-1), while additionally identifying the primary sub-band contributing molecules; and, (ii) sub-bands having primarily single-reference-molecule sourced signal intensity (>90%) could be isolated. A number of reference spectra from basic molecules used in the above-described machine-learning algorithm could be found in Ref. 39 of the main text.

References:

[1]V. Mazet, C. Carteret, D. Brie, J. Idier, and B. Humbert, Background removal from spectra by designing and minimizing a non-quadratic cost function, *Chemom. Intell. Lab. Syst.* **76,** 121-133 (2005).

[2]S. He, W. Zhang, L. Liu, Y. Huang, J. He, W. Xie, P. Wu, and C. Du, Baseline correction for Raman spectra using an improved asymmetric least squares method, *Anal. Methods* **6,** 4402-4407 (2014).

[3]Y. Cai, C. Yang, D. Xu, and W. Gui, Baseline correction for Raman spectra using penalized spline smoothing based on vector transformation, *Anal. Methods* **10,** 3525-3533 (2018).

Supplementary Information

S-1. Labeling of the Raman spectra of viral strains and sub-band assignments

The deconvoluted Raman spectra collected on different viral strain were labeled by numbering their Voigtian sub-bands as shown in Figs. S-1 for JPN/TY/WK-521, hCoV-19/Japan/QK002/2020, and hCoV-19/Japan/QHN001/2020 isolates. Frequencies at maximum, tentative assignments of the vibrational origins of the deconvoluted sub-bands, and related references are listed in Tables S-I(a)~(c) for the frequency intervals 600~750 cm^{-1} (Zone I), 750~900 cm^{-1} (Zone II), 900~1200 cm^{-1} (Zone III), and 1600~1750 cm^{-1} (Zone IV), respectively. Vibrational assignments were given according to literature references as shown in the Tables.

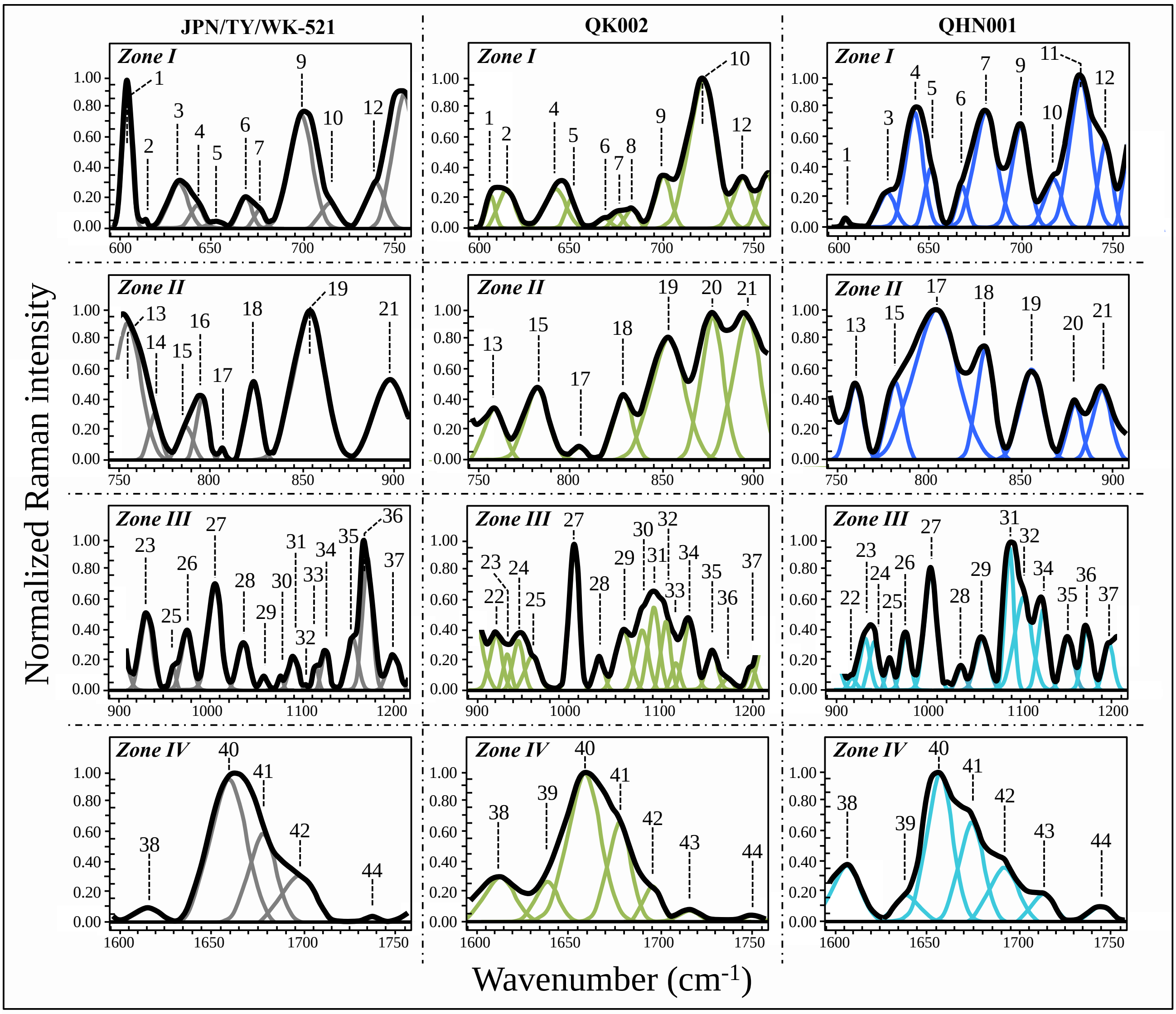

Fig. S-1: Deconvoluted and labeled Raman spectra of different viral strains in different spectral zones. Labels correspond to those in Tables S-I(a)~(d).

Label	cm^{-1}	Assignments	Ref.
1	604	C=O bending in cytosine and uracil	[1]
2	616	COO^{-} rocking in cysteine	[2][3]
3	632	C-S stretching in cysteine	[2]
4	642	C-C-S stretching in methionine (CH_2 side; *gauche*)	[4]
5	652	C-S stretching in methionine (CH_2 side; *gauche*)	[4]
6	669	C-S stretching in methionine (CH_2 side; *trans*)	[4]
7	674	Out-of-plane imidazole ring deformation in adenine	[1]
8	683	C-S stretching in cysteine	[2]
9	698	C-S stretching in methionine (CH_3 side; *gauche*)	[4]
10	719	C-S stretching in methionine (CH_3 side; *trans*)	[4]
11	732	C-S stretching (CH_3 side; *trans*) in high pH	[4]
12	743	In-plane benzene ring breathing in RNA adenine	[1]

Table S-I(a): Frequencies at maximum, vibrational origins, and literature references for sub-bands deconvoluted in Zone I.

Label	cm^{-1}	Assignments	Ref.
13	758	Indole ring breathing in tryptophan	[5]
14	771	Out-of-plane ring deformation in uracil	[1]
15	784	−O−P−O− symmetric stretching in RNA backbone	[6]
16	794	Ring breathing in cytosine<br>Out-of-plane pyrimidine ring deformation in adenine	[1]
17	807	−O−P−O− symmetric stretching in RNA backbone	[6][7]
18	831	Out-of-plane ring vibrations in tyrosine	[3]
19	854	In-plane ring vibrations in tyrosine	[3]
20	878	C-C stretching in hydroxyproline in proteins	[8][9]
21	896	C-C backbone stretching in proteins	[8][9]

Table S-I(b): Frequencies at maximum, vibrational origins, and literature references for sub-bands deconvoluted in Zone II.

Label	cm^{-1}	Assignments	Ref.
22	919	C-C stretching in proline	[10]
23	931	C-C stretching in proteins (α-helix configuration)	[10]
24	949	C-C stretching in amino acids (α-helix configuration)	[10]
25	959	In-plane imidazole ring deformation in RNA guanine	[1]
26	978	C-C stretching in proteins (β-sheet conformation)	[5]
27	1004	Symmetric ring breathing in phenylalanine	[5]
28	1038	C-N-C in-plane ring deformation in cytosine	[1]
29	1054	C-N-C in-plane ring deformation in RNA uracil	[1]
30	1077	Skeletal C-C stretching in lipids	[11]
31	1090	Symmetric phosphate stretching vibrations	[12]
32	1106	Phosphodiester linkages in RNA backbone	[7]
33	1113	C-N stretching in proteins	[13]
34	1125	C-N & C-C skeletal stretching in proteins	[10]
35	1150	C-N ring stretching in RNA adenine	[1]
36	1170	C-H in-plane bending mode of tyrosine	[10]
37	1200	Amide III	[8]

Table S-I(c): Frequencies at maximum, vibrational origins, and literature references for sub-bands deconvoluted in Zone III.

Label	cm^{-1}	Assignments	Ref.
38	1616	In-plane ring vibrations of tyrosine and tryptophan	[3][5]
39	1638	Amide I (β-sheet configuration)	[14]
40	1659	Amide I (α-helix configuration)	[14]
41	1679	Amide I (random coil configuration)	[14]
42	1696	Amide I (β-turn configuration)	[14]
43	1716	Amide I (β-turn configuration)	[14]
44	1749	C=O stretching in phospholipids	[11]

Table S-I(d): Frequencies at maximum, vibrational origins, and literature references for sub-bands deconvoluted in Zone IV.

S-2. Comparison with genome data

A comparison between fractions of purine and pyrimidine bases computed from the Raman spectrum and from genome analysis [15] is shown in Table S-II.

Base	TY-WK-521		QH002		QHN002	
	R	***G***	***R***	***G***	***R***	***G***
Adenine	37.8%	29.9%	34.3%	29.9%	23.9%	29.9%
Cytosine	35.5%	18.4%	14.8%	18.4%	18.3%	18.3%
Guanine	15.5%	19.6%	18.5%	19.6%	21.1%	19.6%
Uracil	11.2%	32.1%	32.4%	32.2%	36.7%	32.1%

Table S-II: Comparison between fractions of purine and pyrimidine fractions computed from Raman (R) and genome (G) analyses in different isolates.

S-3. Statistical analysis of Raman spectra according to the Pearson's correlation coefficient

The so-called Pearson's correlation coefficient (PC), r, also referred to as spectral similarity coefficient, can be calculated according to following equation [16]:

$$r = \frac{\sum_{i=1}^{n} Q_i s_i - \frac{1}{n}\left(\sum_{i=1}^{n} Q_i \sum_{i=1}^{n} s_i\right)}{\sqrt{\left[\sum_{i=1}^{n} Q_i^2 - \frac{1}{n}\left(\sum_{i=1}^{n} Q_i\right)^2\right]\left[\sum_{i=1}^{n} s_i^2 - \frac{1}{n}\left(\sum_{i=1}^{n} s_i\right)^2\right]}} \qquad \text{(s-1)}$$

where Q represents an average spectrum from a given variant/sub-type in the database, s is the spectrum to be assessed, and n is the total number of CCD pixels in each of the two spectra. Note that the wavenumber interval to be compared and the pixel intervals should be exactly the same. A perfect matching or a complete mismatching between the database average spectrum Q and the spectrum under assessment s give r =1 and r = 0, respectively. PC values and related standard deviations, as calculated according to the above Eq. (s-1) are listed in Table S-III.

	JPN/TY/WK-521	QHN001	QK002
JPN/TY/WK-521	0.925 ± 0.065	0.386 ± 0.120	0.366 ± 0.043
QHN001	0.485 ± 0.140	0.985 ± 0.027	0.983 ± 0.246
QK002	0.416 ± 0.084	0.976 ± 0.214	0.996 ± 0.001

Table S-III: Cross comparison of r values among variants and sub-types confirming almost identical patterns ($r > 0.920$) only when comparing spectra collected on samples of the same variant/sub-type (cf. values along the table diagonal), while a low PC score ($r < 0.420$) is found by comparing spectra from different variants/sub-types.

S-4. Comparison of Raman spectra collected at different locations

Comparisons between average Raman spectra and spectra collected at individual locations on the same variant/sub-type are shown hereafter (Fig. S-2 ~ Fig. S-11). The figures include sub-band-deconvolution analyses for fractions of sulfur-containing amino acid rotamers, tyrosine phenol ring, apparent fractions of RNA purines and pyrimidines, and protein secondary structures. Individual spectra collected at different locations generally showed agreement with the corresponding average one in agreement with Pearson's statistical assessments (in Table S-III). However, some interesting variations in local spectra could be found. Although lacking statistical significance, such anomalous spectra, could unveil additional structural features.

Looking, for example, at the morphology of the tyrosine doublet in average spectra, namely, the I_{854}/I_{826} ratio as a sensor of hydrophobic/hydrophilic balance at the virion surface, the QK002 sub-type of the British variant experienced in average a high I_{854}/I_{826} ratio (~1.8; cf. Figs. 3 and S-9(a)). This is a label for a hydrophilic tyrosine configuration and reveals an acidic environment at the virion surface experienced by the majority of the virions belonging to this species (cf. Figs. S-9(a) and (c)). However, some anomalous locations in the QK002 sample showed the opposite trend (cf. Fig. S-9(c)), which indeed proves the presence of some inhomogeneity in the virions' population. Interestingly, the anomalous QK002 locations with an I_{854}/I_{826} ratio <1 also presented a different distribution of protein secondary structures, with a clearly larger relative fraction of random coil configuration (cf. sub-band displaying at ~1676 cm^{-1} in Fig. S-11(c)), as compared to α-helix. A similar trend could be observed for the original Japanese isolate JPN/TY/WK-521 (cf. Figs. S-4(c) and S-6(c)). However, a peculiar anomaly in the spectrum of the original isolate consisted in the presence of a tyrosinate band at 836~840 cm^{-1}. This anomaly was never found in any of the two

British variant's sub-types. The anionic form of tyrosine is expected at high values of interfacial pH with tyrosine ratio, namely, only in spectra with tyrosine ratios >1. However, this was not always the case (cf. Figs. S-4(b) and (c)). The presence of the ~840 cm^{-1} tyrosinate sub-band in anomalous spectra with tyrosine ratios <1 indeed represents a confirmation of the higher heterogeneity of the original Japanese isolate, since it can only be explained by the concurrent presence of different virion populations in the same sample.

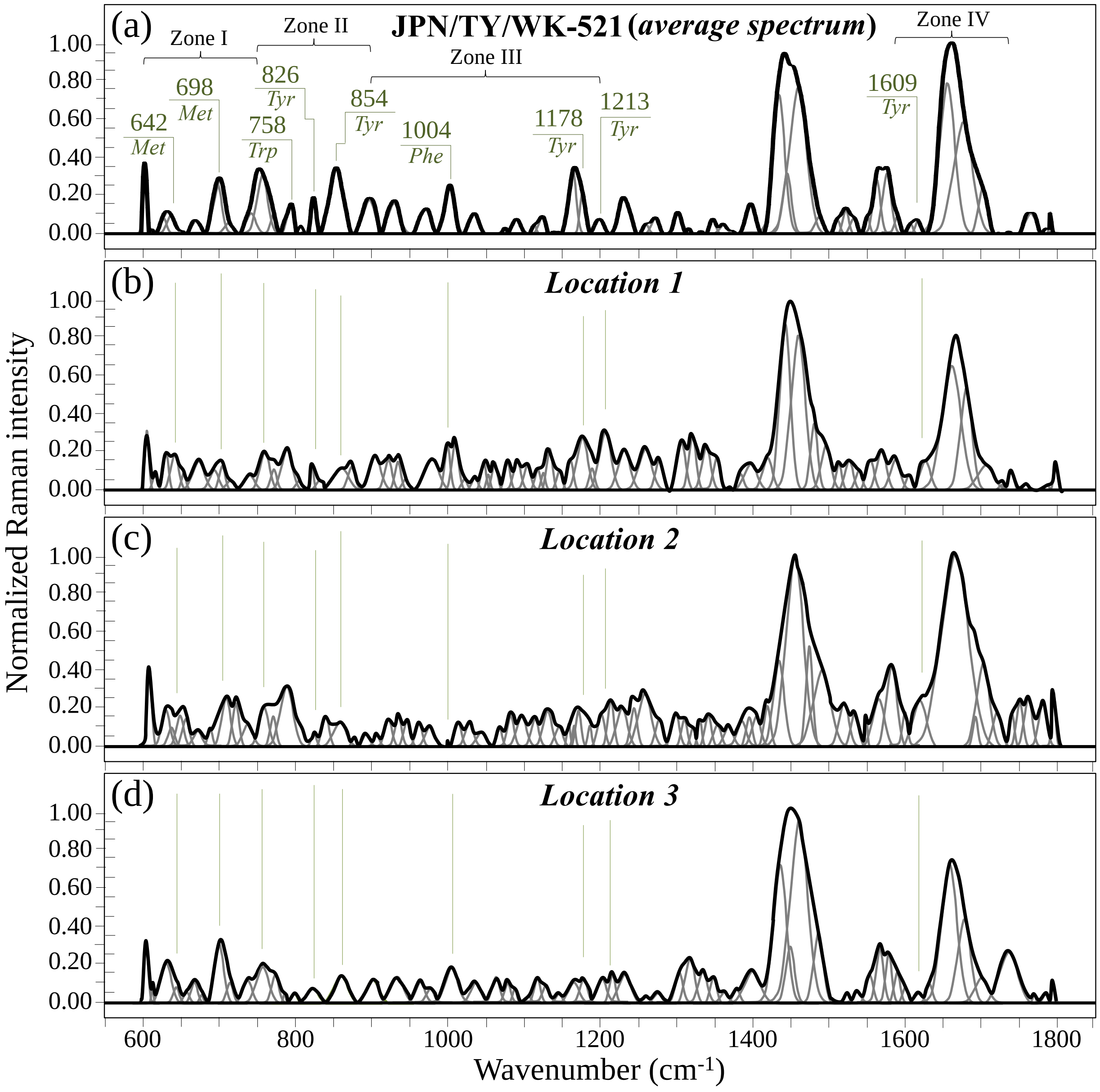

Fig. S-2: Average Raman spectrum of the original Japanese isolate JPN/TY/WK-521 as collected at different locations in the spectral zone 600~1800 cm^{-1} (a) is compared with individual spectra collected in the same spectral interval at three different locations on the same sample ((b), (c), and (d) for Locations 1, 2 and 3, respectively).

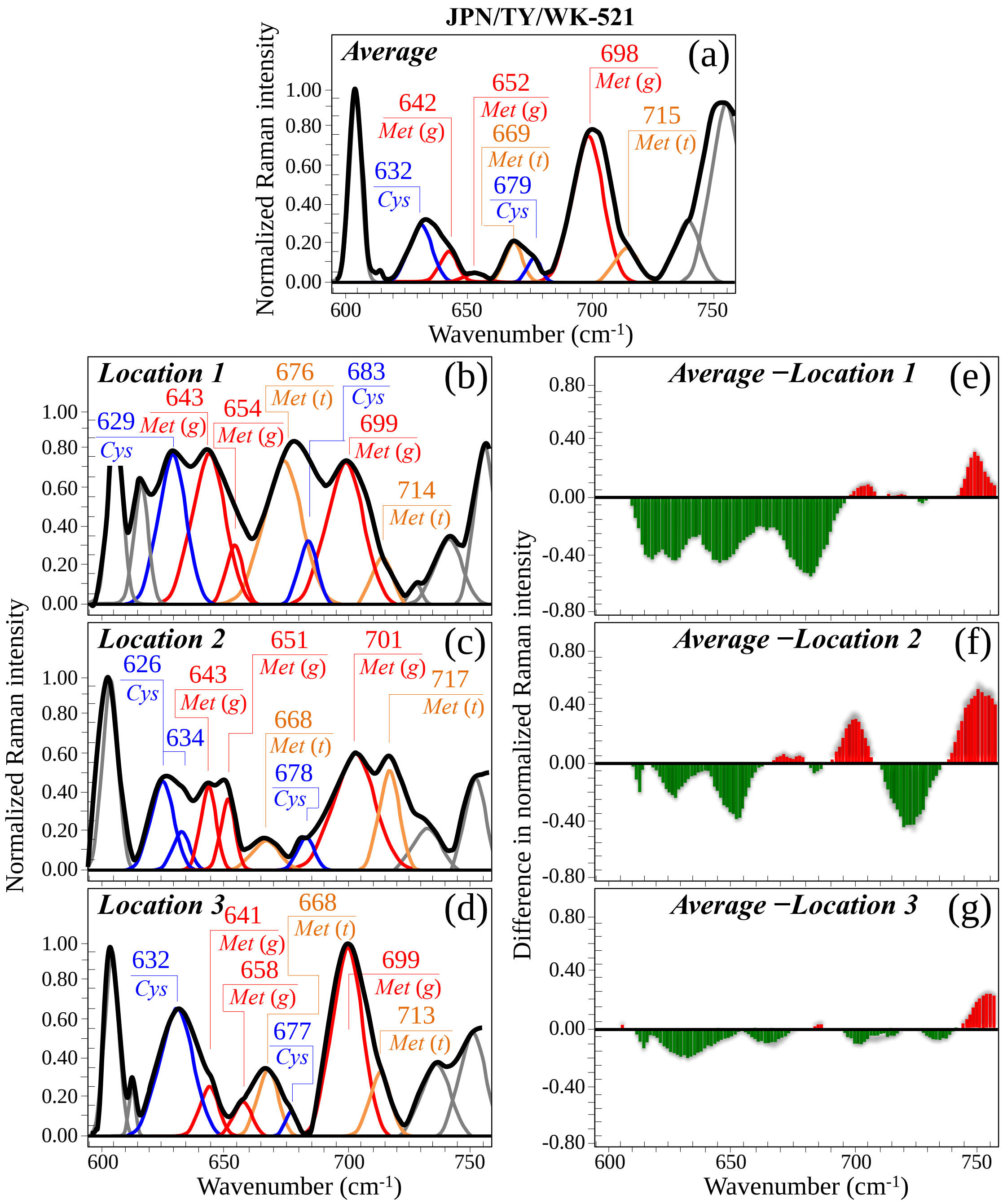

Fig. S-3: Deconvoluted and labeled Raman spectra from the original Japanese isolate JPN/TY/WK-521 as collected at different locations in the spectral zone 600~750 cm^{-1}: (a) Average spectrum as given in Fig. 2(a) of the paper; (b), (c), and (d) spectra collected at three different locations; and, (e), (f), and (g) difference in Raman intensity between the average spectrum and spectra in (b), (c), and (d), respectively. In the labels, the wavenumbers of individual sub-bands are given in cm^{-1}. The abbreviations *Met* and *Cys* refer to methionine and cysteine, respectively; (*t*) and (*g*) refer to trans and gauche rotamers.

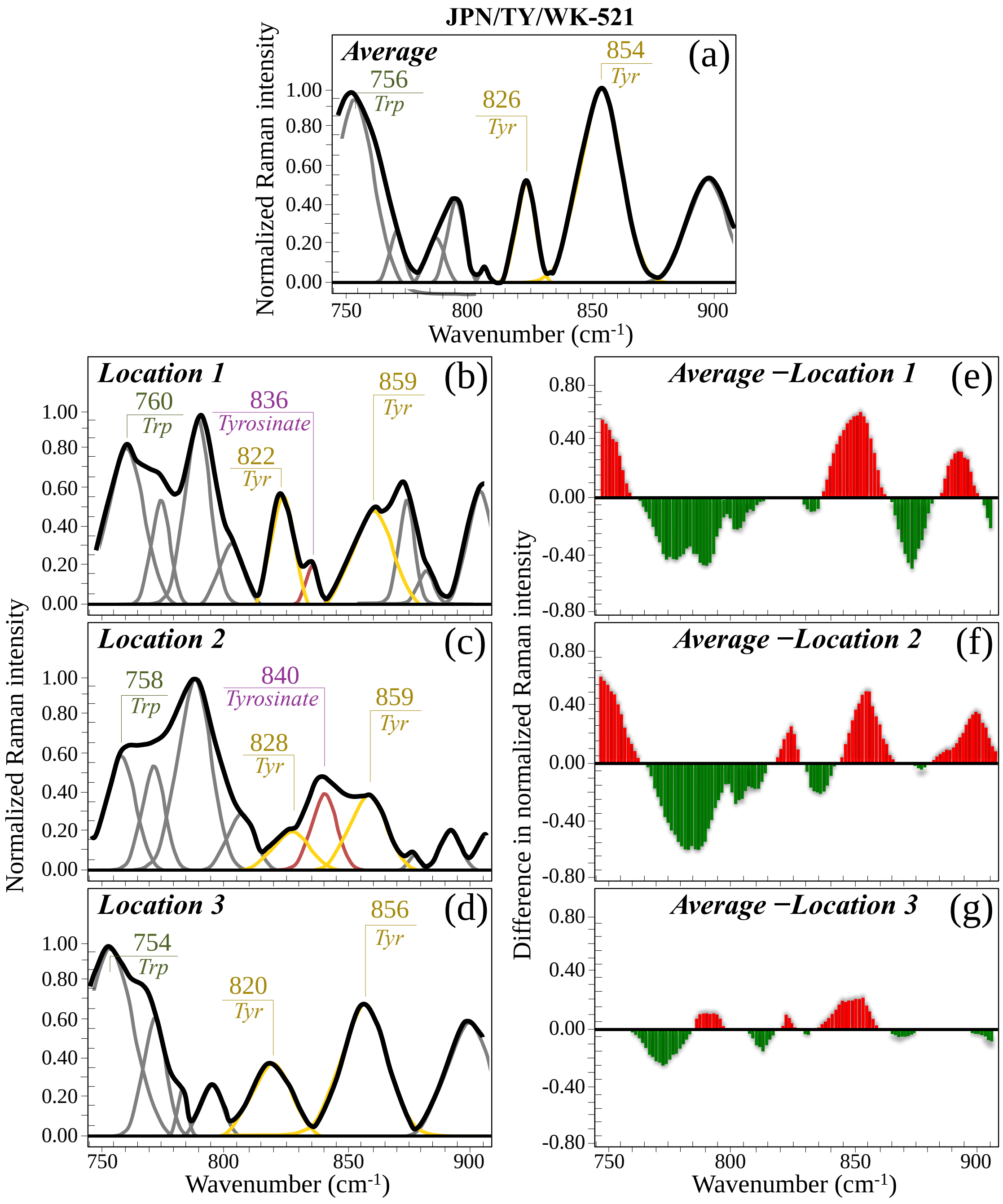

Fig. S-4: Deconvoluted and labeled Raman spectra from the original Japanese isolate JPN/TY/WK-521 as collected at different locations in the spectral zone 750~900 cm^{-1}: (a) Average spectrum as given in Fig. 3(a) of the paper; (b), (c), and (d) spectra collected at three different locations; and, (e), (f), and (g) difference in Raman intensity between the average spectrum and spectra in (b), (c), and (d), respectively. In the labels, the wavenumbers of individual sub-bands are given in cm^{-1}. The abbreviations *Trp* and *Tyr* refer to tryptophan and tyrosine, respectively; tyrosinate is the anionic form of tyrosine.

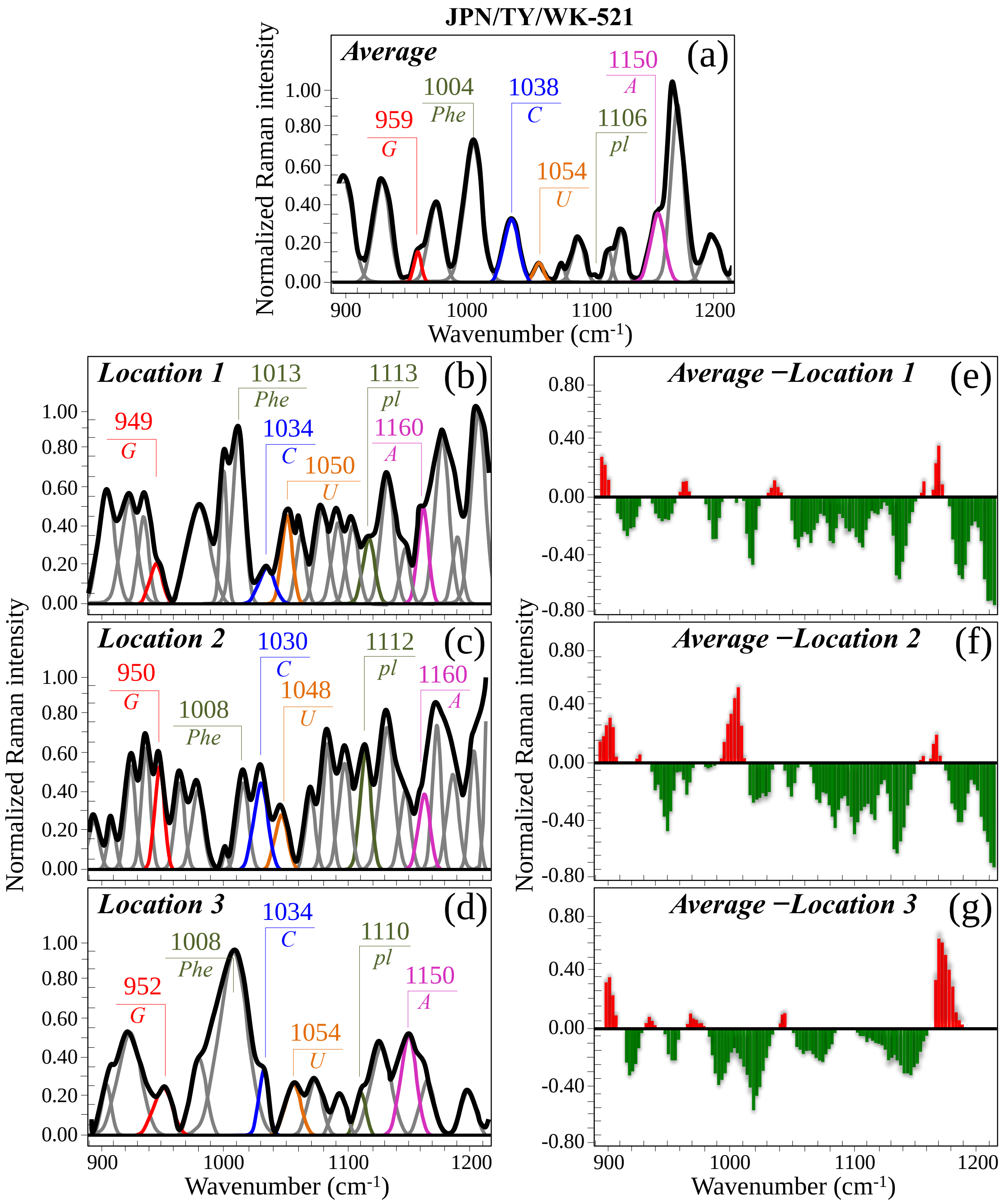

Fig. S-5: Deconvoluted and labeled Raman spectra from the original Japanese isolate JPN/TY/WK-521 as collected at different locations in the spectral zone 900~1200 cm^{-1}: (a) Average spectrum as given in Fig. 4(a) of the paper; (b), (c), and (d) spectra collected at three different locations; and, (e), (f), and (g) difference in Raman intensity between the average spectrum and spectra in (b), (c), and (d), respectively. In the labels, the wavenumbers of individual sub-bands are given in cm^{-1}. The abbreviations *G*, *Phe*, *C*, *U*, *pl* and *A* refer to guanine, phenylalanine, cytosine, uracil, phosphodiester linkages, and adenine, respectively.

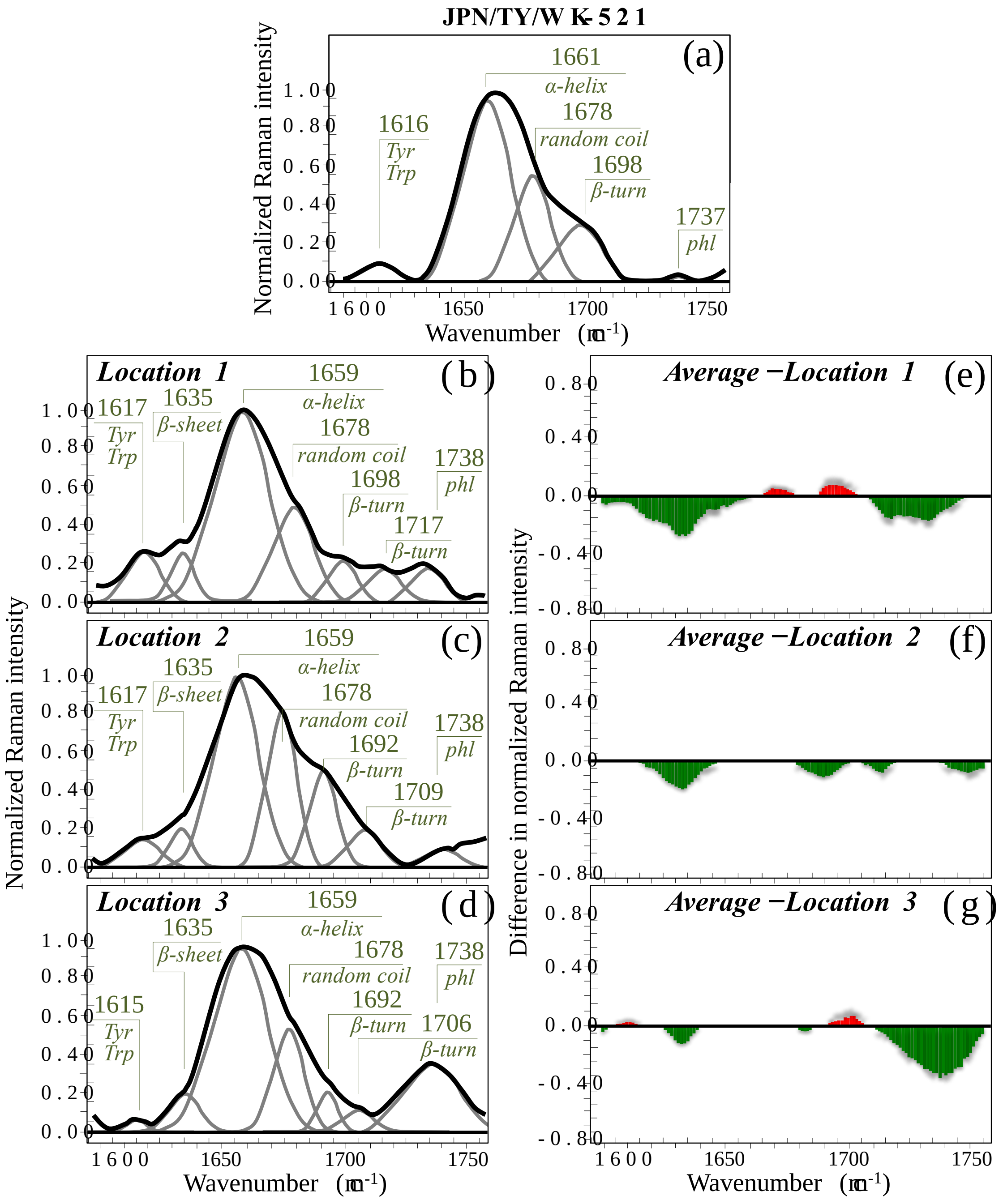

Fig. S-6: Deconvoluted and labeled Raman spectra from the original Japanese isolate JPN/TY/WK-521 as collected at different locations in the spectral zone 1600~1750 cm^{-1}: (a) Average spectrum as given in Fig. 5(a) of the paper; (b), (c), and (d) spectra collected at three different locations; and, (e), (f), and (g) difference in Raman intensity between the average spectrum and spectra in (b), (c), and (d), respectively. In the labels, the wavenumbers of individual sub-bands are given in cm^{-1}.

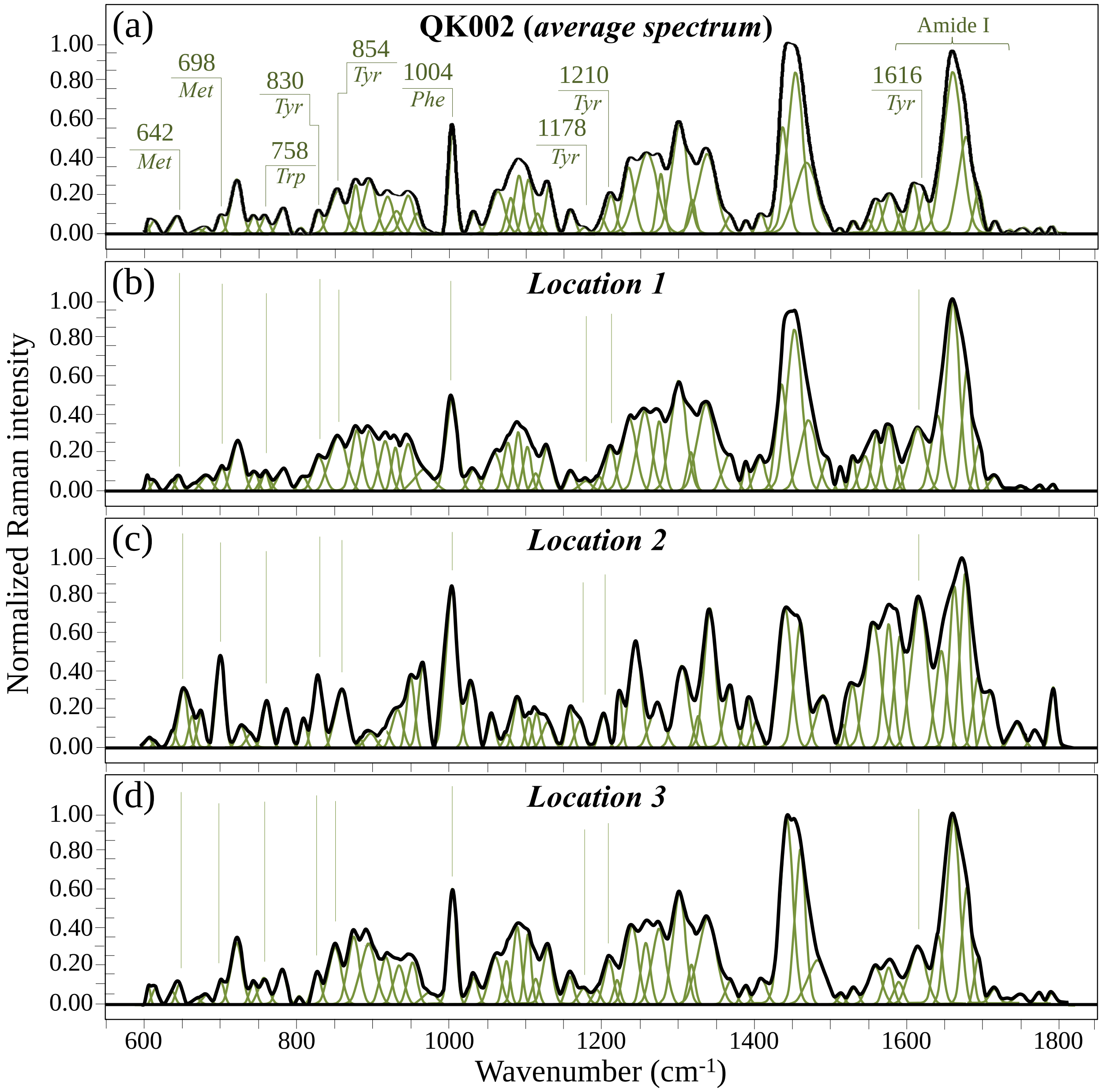

Fig. S-7: Average Raman spectrum of the sub-type QK002 of the British variant as collected at different locations in the spectral zone 600~1800 cm^{-1} (a) is compared with individual spectra collected in the same spectral interval at three different locations on the same sample ((b), (c), and (d) for Locations 1, 2 and 3, respectively).

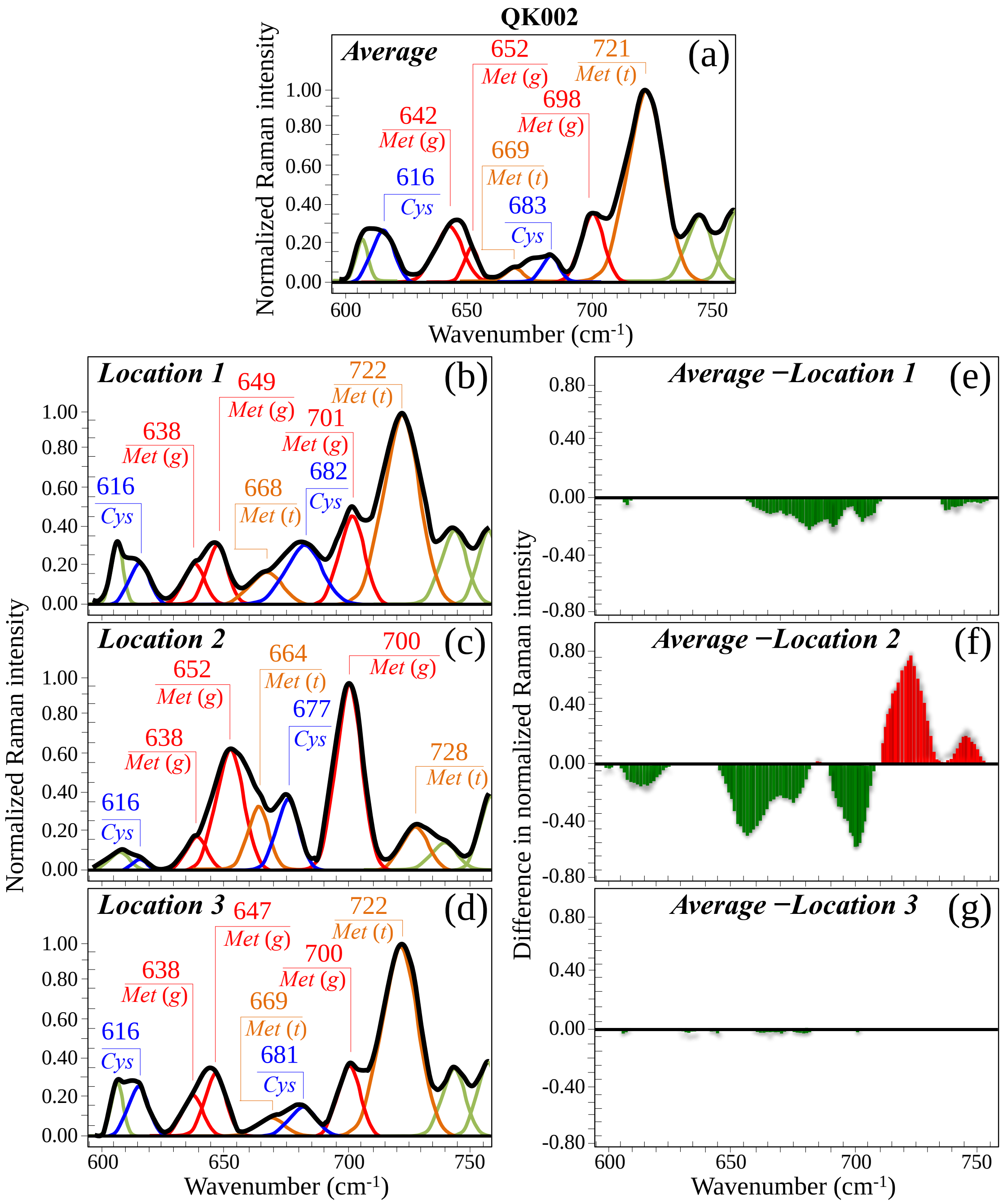

Fig. S-8: Deconvoluted and labeled Raman spectra from the sub-type QK002 of the British variant as collected at different locations in the spectral zone 600~750 cm^{-1}: (a) Average spectrum as given in Fig. 2(b) of the paper; (b), (c), and (d) spectra collected at three different locations; and, (e), (f), and (g) difference in Raman intensity between the average spectrum and spectra in (b), (c), and (d), respectively. In the labels, the wavenumbers of individual sub-bands are given in cm^{-1}. The abbreviations *Met* and *Cys* refer to methionine and cysteine, respectively; (*t*) and (*g*) refer to trans and gauche rotamers.

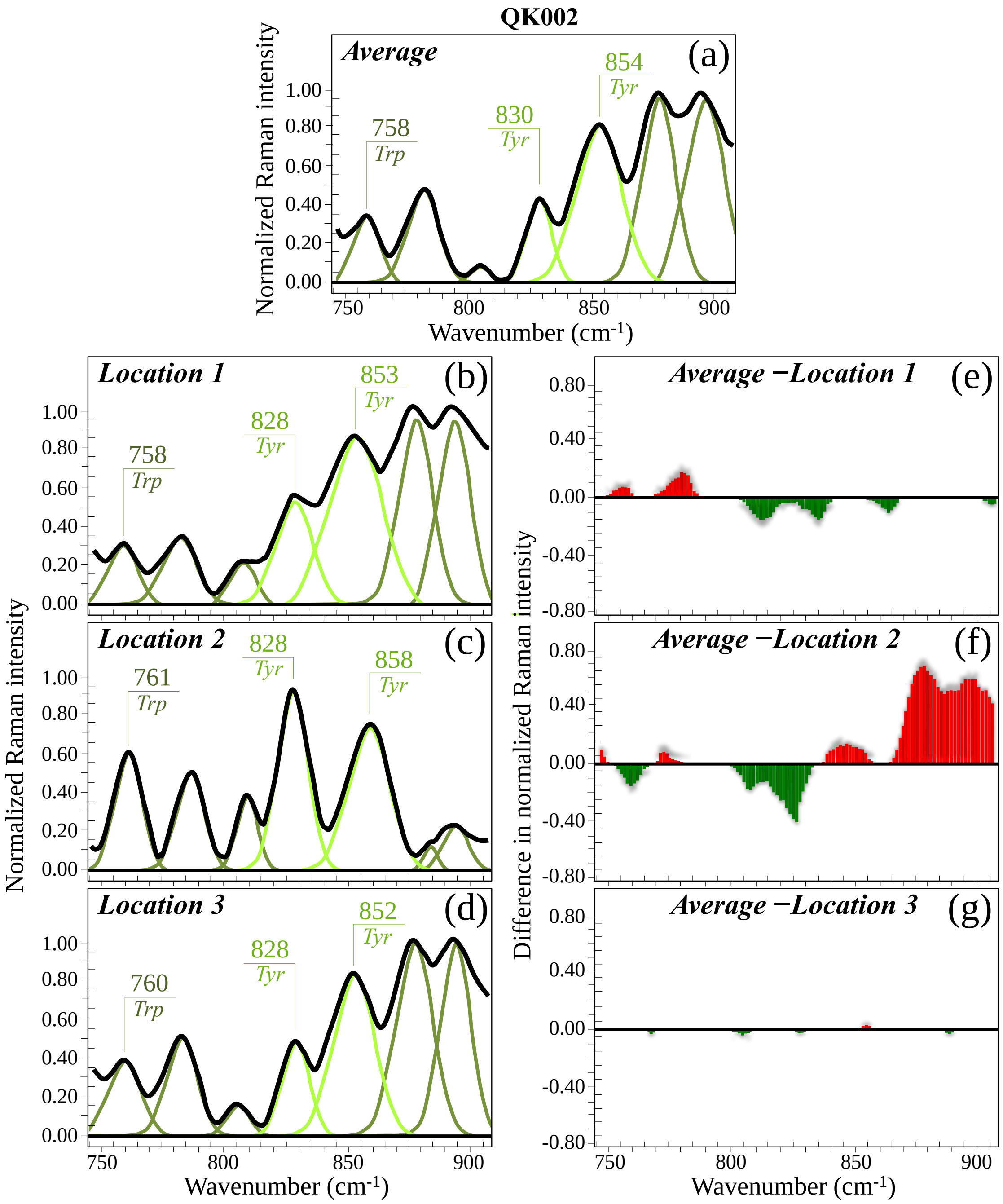

Fig. S-9: Deconvoluted and labeled Raman spectra from the sub-type QK002 of the British variant as collected at different locations in the spectral zone 750~900 cm^{-1}: (a) Average spectrum as given in Fig. 3(b) of the paper; (b), (c), and (d) spectra collected at three different locations; and, (e), (f), and (g) difference in Raman intensity between the average spectrum and spectra in (b), (c), and (d), respectively. In the labels, the wavenumbers of individual sub-bands are given in cm^{-1}. The abbreviations *Trp* and *Tyr* refer to tryptophan and tyrosine, respectively; tyrosinate is the anionic form of tyrosine.

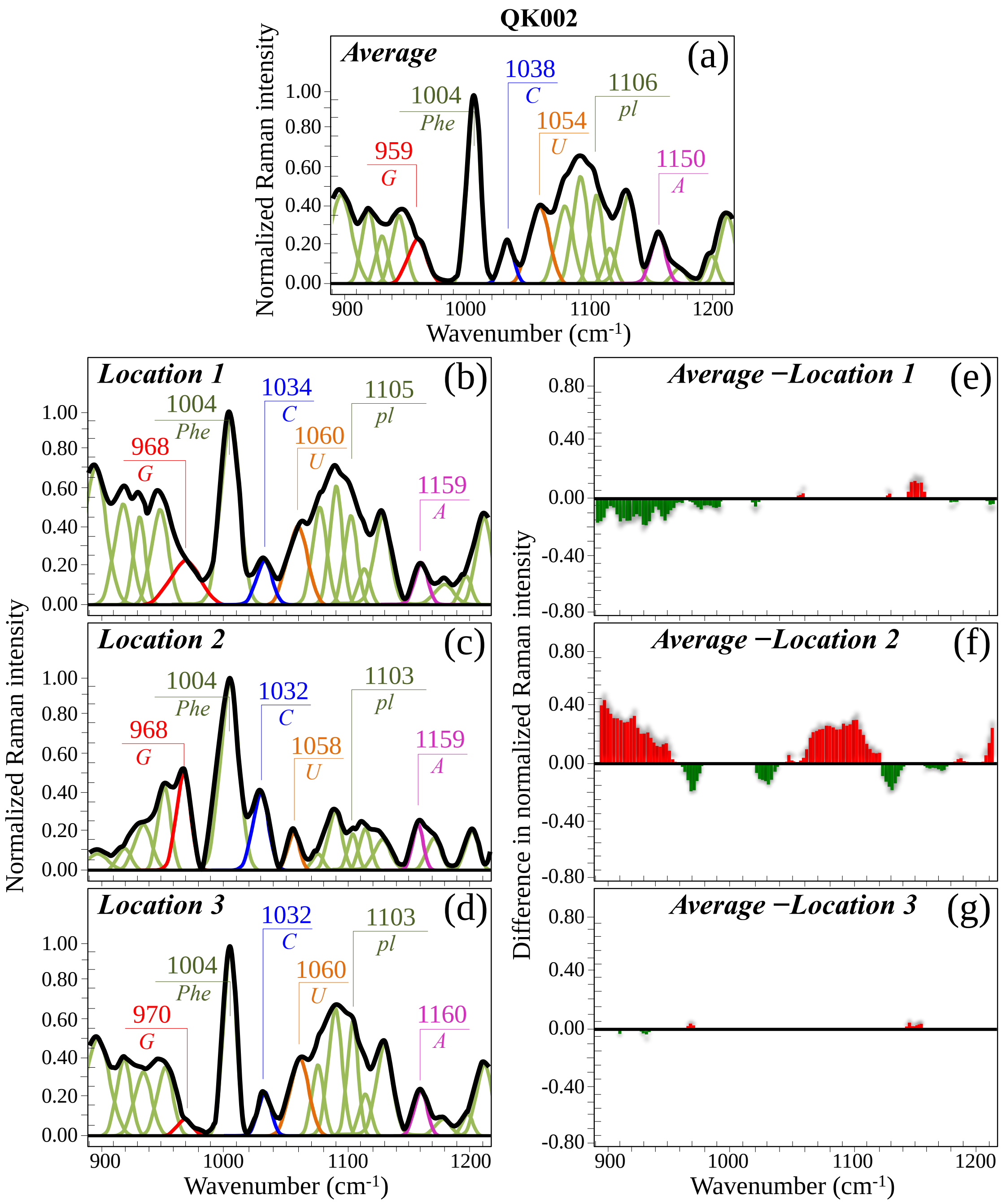

Fig. S-10: Deconvoluted and labeled Raman spectra from the sub-type QK002 of the British variant as collected at different locations in the spectral zone 900~1200 cm^{-1}: (a) Average spectrum as given in Fig. 4(b) of the paper; (b), (c), and (d) spectra collected at three different locations; and, (e), (f), and (g) difference in Raman intensity between the average spectrum and spectra in (b), (c), and (d), respectively. In the labels, the wavenumbers of individual sub-bands are given in cm^{-1}. The abbreviations *G*, *Phe*, *C*, *U*, *pl* and *A* refer to guanine, phenylalanine, cytosine, uracil, phosphodiester linkages, and adenine, respectively.

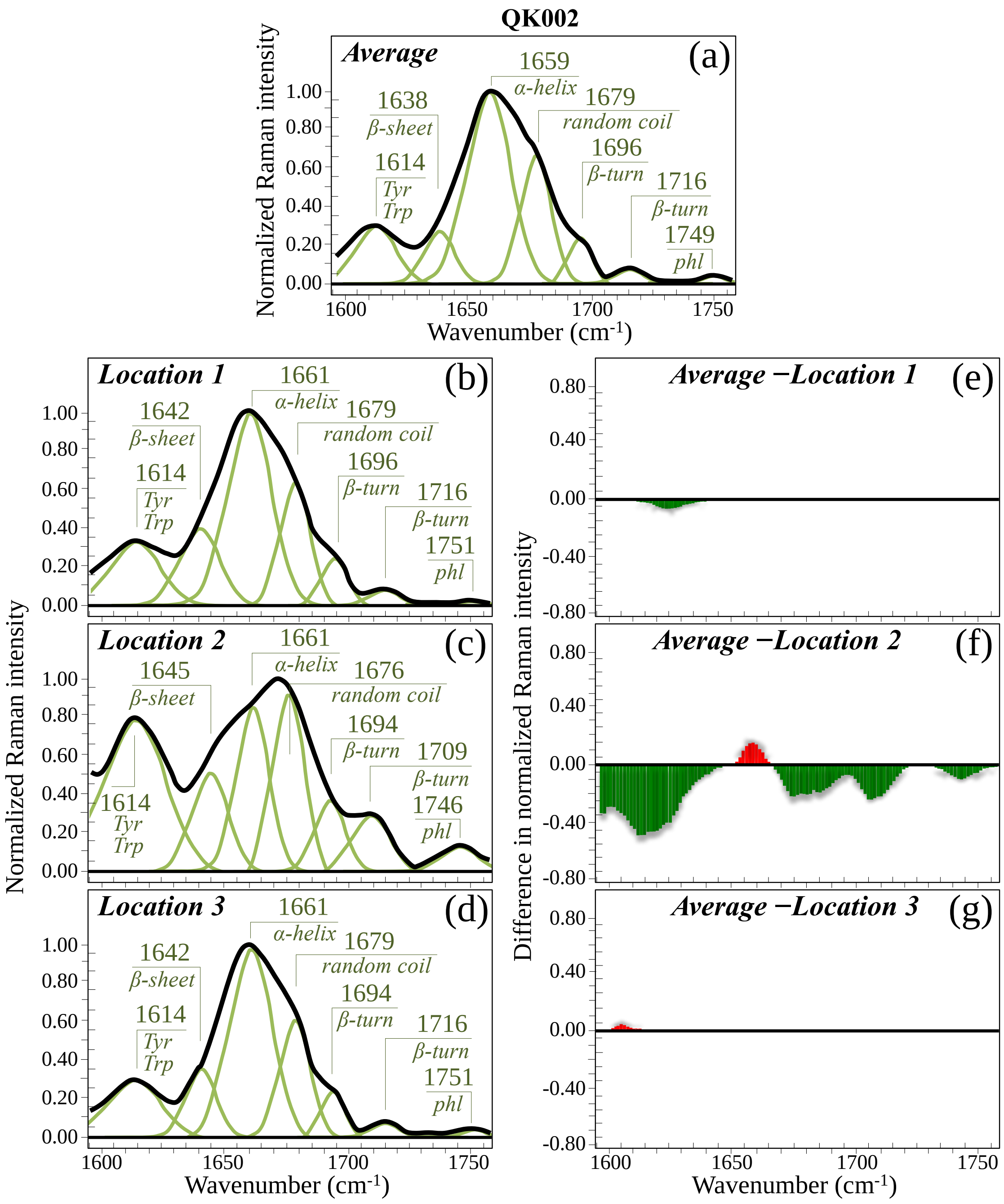

Fig. S-11: Deconvoluted and labeled Raman spectra from the sub-type QK002 of the British variant as collected at different locations in the spectral zone 1600~1750 cm^{-1}: (a) Average spectrum as given in Fig. 5(b) of the paper; (b), (c), and (d) spectra collected at three different locations; and, (e), (f), and (g) difference in Raman intensity between the average spectrum and spectra in (b), (c), and (d), respectively. In the labels, the wavenumbers of individual sub-bands are given in cm^{-1}.

References:

[1] F. Madzharova, Z. Heiner, M. Guehlke, and J. Kneipp, *J. Phys. Chem.* **120,** 15415 (2016).

[2] P. Bazylewski, R. Divigalpitiya, and G. Fanchini, *RSC Adv.* **7,** 2964 (2017).

[3] B. Hernandez, F. Pfluger, A. Adenier, S. K. Kruglik, and M. Ghomi, *Phys. Chem. Chem. Phys.* **13,** 17284 (2011).

[4] E. Podstawka, Y. Ozaki, and L. M. Proniewicz, *Appl. Spectrosc.* **58,** 581 (2004).

[5] G. Zhu, X. Zhu, Q. Fan, X. Wan, *Spectrochim. Acta Part A* **78,** 1187 (2011).

[6] D. Naumann, *Proc. SPIE* **3257,** 245 (1998).

[7] G. J. Thomas, Jr., and K. A. Hartman, *Biochim. Biophys. Acta* **312,** 311 (1973).

[8] J. W. Chan, D. S. Taylor, T. Zwerdling, S. T. Lane, K. Ihara, and T. Huser, *Biophys. J.* **90,** 648 (2006).

[9] C. J. Frank, R. L. McCreecy, and D. C. B. Redd, *Anal. Chem.* **67,** 777 (1995).

[10] N. Stone, C. Kendall, J. Smith, P. Crow, H. and Barr, *Faraday Discussion* **126,** 141 (2004).

[11] C. Krafft, L. Neudert, T. Simat, R. Salzer, *Spectrochim. Acta Part I* **61,** 1529 (2005).

[12] R. K. Dukor, *Biomed. Appl.* **5,** 3335 (2002).

[13] Z. Huang, A. McWilliams, M. Lui, D. I. McLean, S. Lam, and H. Zeng, *Int. J. Cancer* **107,** 1047 (2003).

[14] M. Voicescu, S. Ionescu, C. L. Nistor, *Spectrochim. Acta Part A: Mol. Biomol. Spectrosc.* **170,** 1 (2017).

[15] https://www.gisaid.org/

[16] A. Z. Samuel, R. Mukojima, S. Horii, M. Ando, S. Egashira, T. Nakashima, M. Iwatsuki, and H. Takeyama, *ACS Omega* **6,** 2060 (2021).
